# The results of prolonged administration of isoniazid to mice, rats and hamsters.

**DOI:** 10.1038/bjc.1966.39

**Published:** 1966-06

**Authors:** A. Peacock, P. R. Peacock

## Abstract

**Images:**


### THE RESULTS OF PROLONGED ADMINISTRATION OF ISONIAZID TO MICE, RATS AND HAMSTERS

ANDRÉE PEACOCK* AND P. R. PEACOCK

*From the Cancer Research Department, Royal Beatson Memorial Hospital, Glasgow*

Received for publication, January 28, 1966

THE demonstration that isoniazid (INH) is carcinogenic for the lungs of mice (Juhasz, Balò and Kendrey, 1957) raised a new type of cancer problem. Most known carcinogens are avoidable as human hazards and many are subject to restrictive legislation. However, INH is a valuable life-saving drug in the control of tuberculosis, and the assessment of it as a potential carcinogenic hazard for man is therefore a matter of unusual concern.

Moreover, it must be noted that the induction of pulmonary alveolar adenoma or adenocarcinoma in mice by a variety of chemically dissimilar carcinogens (e.g. urethane and 4-nitro-quinoline-N-oxide) is most striking in those strains, such as A and BALB/c, which have a high spontaneous incidence of such tumours. Tumours of similar histogenesis can be induced experimentally in rats but they very rarely occur spontaneously. In terms of initiators and promoters, mice of different strains behave as though they have variable amounts of some inborn initiator which determines the response of their alveolar cells to a variety of carcinogenic promoters. Apart from known genetic factors no "initiator" as postulated has been identified.

It seemed desirable, therefore, to assay INH on animals other than mice, under comparable conditions of exposure, and at non-toxic dosage, for the major part of their lives.

### MATERIAL AND METHODS

All rodents were fed on Diet No. 41 pellets.

Pilot experiments showed that with our strains of C3Hf mice and BALB/c mice a single subcutaneous injection of 2 mg. or a daily oral intake of 2 mg. INH per adult mouse of about 20 g. was toxic for about a third of the animals, but was tolerated when given by gastric intubation not more frequently than twice a week, or daily in drinking water probably because in the latter case the drug was taken gradually over 24 hours.

### *Subcutaneous injection*

Mice—Thirty male and 10 female C3Hf aged between 5–17 weeks and 23 male and 16 female BALB/c mice aged between 7–36 weeks were injected subcutaneously with 0·2 ml. of INH solution equivalent to 2 mg. per injection.

At first injections were given three times a week, but owing to heavy mortality and evidence of peripheral neuritis in the survivors, the frequency of injection was reduced. Thus those which died early in the experiment received relatively

* Working under a full time grant from the British Empire Cancer Campaign for Research.

higher total dosage. The total dose varied somewhat between mice, because those which looked sick were not injected until they had recovered. Over a period of 8 months 56 injections were given.

*Results.*—No pulmonary tumours were seen in mice which died at less than 40 weeks of age. Of C3Hf mice that survived beyond this age, of 17 males only 1, which died aged 36 weeks, had a deep-seated papillary adenoma ; no pulmonary tumours occurred in 6 female survivors (Table I).

TABLE I.—*INH Subcutaneous Injections—C3Hf Mice*

| No. | Age in weeks at start | Age in weeks at death | Period of injection (weeks) | INH Total dose (mg.) | Lung 1 | Lung 2 | Lung 3 | Lung 4 | Autopsy and histological diagnosis |
|---|---|---|---|---|---|---|---|---|---|
| Males | | | | | | | | | |
| 1144/9 | 8 | 8 | 1 day | 2 | .. | .. | .. | .. | Acute toxaemia. |
| 1144/10 | 8 | 8 | 1 day | 2 | .. | .. | .. | .. | „ „ |
| 1166/57 | 8 | 8 | 1 day | 2 | .. | .. | .. | .. | Haemorrhagic enteritis. |
| 1143/5 | 8 | 9 | 1 | 4 | .. | .. | .. | .. | Toxaemia. |
| 1143/6 | 8 | 9 | 1 | 4 | .. | .. | .. | .. | „ |
| 1143/4 | 8 | 10 | 2 | 8 | .. | .. | .. | .. | Lung congested. Desquamation of bronchial epithelium. |
| 1144/11 | 8 | 10 | 2 | 8 | .. | .. | .. | .. | Toxaemia ; no gross lesions. |
| 1144/13 | 8 | 10 | 2 | 8 | .. | .. | .. | .. | „ „ „ „ |
| 1143/45 | 8 | 14 | 6 | 16 | .. | .. | .. | .. | „ „ „ „ |
| 1146/17 | 8 | 14 | 6 | 24 | +* | .. | .. | .. | ?Tumour R.A. lobe.* |
| 1146/20 | 8 | 14 | 6 | 20 | .. | .. | .. | .. | No gross lung lesions. Urethra blocked by vesical calculus. |
| 1146/19 | 8 | 15 | 7 | 24 | .. | .. | .. | .. | No gross lesions. |
| 1166/60 | 8 | 17 | 9 | 30 | .. | .. | .. | .. | Lungs congested. Peribronchial lymphangitis. |
| 1165/55 | 8 | 17 | 9 | 30 | .. | .. | .. | .. | Lungs congested. |
| 1165/52 | 8 | 29 | 21 | 60 | .. | .. | .. | .. | Lungs congested. |
| 1160/27 | 21 | 30 | 9 | 22 | .. | .. | .. | .. | No gross lesions. |
| 1146/15 | 8 | 35 | 27 | 80 | .. | .. | .. | .. | Lungs and kidneys acutely congested. |
| 1182/71 | 8 | 36 | 25 | 88 | .. | + | .. | .. | Congested lung. Papillary adenoma of hilum ; traumatic obstruction of penis ; retention of urine. |
| 1166/63 | 8 | 46 | 30 | 96 | .. | .. | .. | .. | No gross lesions. |
| 1160/30 | 21 | 52 | 31 | 64 | .. | .. | .. | .. | No gross lesions in lungs. Neck broken by cage. |
| 1146/16 | 8 | 63 | 36 | 116 | .. | .. | .. | .. | Acute congestion of lung. |
| 1160/29 | 21 | 66 K | 35 | 102 | .. | .. | .. | .. | No gross lesions in lungs. |
| 1182/68 | 8 | 68 K | 25 | 88 | .. | .. | .. | .. | Lungs congested. |
| 1182/70 | 8 | 68 K | 25 | 88 | .. | .. | .. | .. | „ „ |
| 1182/72 | 8 | 68 K | 25 | 88 | .. | .. | .. | .. | „ „ |
| 1165/51 | 8 | 69 K | 30 | 96 | .. | .. | .. | .. | Congested lungs and kidneys. |
| 1166/59 | 8 | 69 K | 30 | 96 | .. | .. | .. | .. | No gross lesions in lungs. |
| 1143/7 | 8 | 73 K | 36 | 116 | .. | .. | .. | .. | ? Tumour R.M. lobe.* Subpleural oedema and lymphangitis. |
| 1143/2 | 8 | 74 | 36 | 116 | .. | .. | .. | .. | No gross lesions. |
| 1182/69 | 8 | 92 | 25 | 88 | .. | .. | .. | .. | Lungs congested. |
| Females | | | | | | | | | |
| 1181/64 | 8 | 8 | 1 day | 2 | .. | .. | .. | .. | Acute toxaemia. |
| 1181/65 | 8 | 8 | 1 day | 2 | .. | .. | .. | .. | „ „ |
| 1181/66 | 8 | 8 | 1 day | 2 | .. | .. | .. | .. | „ „ |
| 1181/67 | 8 | 12 | 2 | 4 | .. | .. | .. | .. | Patchy congestion. |
| 1183/77 | 8 | 16 | 6 | 11 | .. | .. | .. | .. | No gross lesions in lung. |
| 1183/76 | 8 | 22 | 12 | 16 | .. | .. | .. | .. | „ „ „ „ „ |

TABLE I. (*contd*)

| No. | Age in weeks at start | Age in weeks at death | Period of injection (weeks) | INH Total dose (mg.) | Lung 1 | Lung 2 | Lung 3 | Lung 4 | Autopsy and histological diagnosis |
|---|---|---|---|---|---|---|---|---|---|
| 1183/74 | 8 | 28 | 17 | 25 | .. | .. | .. | .. | Lung: slight alveolar exudate and lymphocytosis. |
| 1181/63 | 8 | 28 | 17 | 25 | .. | .. | .. | .. | Lung congested; fibrinous exudate in alveoli. |
| 1183/73 | 8 | 29 | 21 | 37 | .. | .. | .. | .. | White spots R.U. and R.L. Lung congested. |
| 1183/75 | 8 | 68 K | 26 | 44 | .. | .. | .. | .. | No gross lesions. |

The following abbreviations have been used throughout the tables :
K = Killed.
* = Not histologically confirmed.
Lung : Column 1 = Papillary adenoma of subpleural origin.
2 = Papillary adenoma of other sites.
3 = Alveolar hyperplasia of subpleural origin.
4 = Alveolar hyperplasia of other sites.
Lobes of Lung : R.U. = Right upper.
R.M. = Right middle.
R.L. = Right lower.
R.A. = Right azygos.
L. = Left.

Among male BALB/c mice which survived for more than 40 weeks, 8 out of 19 had no pulmonary tumours. In the remaining 11 there were 8 with subpleural papillary adenomata, 6 with papillary adenomata at other sites, 1 with hyperplasia at subpleural and at other sites.

Among 11 females, over 40 weeks old, 3 had no pulmonary tumours. Among 8 with lung tumours, 6 had subpleural papillary adenomata at other sites and 3 had alveolar hyperplasia at other sites and 1 at subpleural and at other sites (Table II).

*Gastric intubation*

Twenty-four male and 17 female BALB/c mice were given INH by gastric intubation three times a week, or less frequently if toxic effects appeared, for 40 to 45 weeks. A daily dose of 2 mg. caused some early deaths due to toxicity of the drug. Sixteen males and 11 females survived for more than 40 weeks, but all died or were killed by 65 weeks. The survival, duration of exposure and total dose of INH and site and type of tumour are shown in Table III.

*Results*.—One subpleural and one deep-seated papillary adenoma in a male and one subpleural tumour, not confirmed histologically, and one subpleural papillary adenoma in a female were found ; all others were free of pulmonary tumours, but most of them had chronic respiratory lesions, septic bronchitis, bronchopneumonia, or bronchiectasis.

It was found impossible to distinguish infected tumours from peripheral bronchiectatic abscesses on naked eye examination and only microscopic examination of serial sections revealed the true character of different lesions (Fig. 1 and 2).

It was felt that daily administration without instrumentation of any kind was preferable because it resembled the method of administration to human patients. Metabolic studies (Chalmers, 1965) showed that the drug was absorbed,

TABLE II.—*INH Subcutaneous Injections—BALB/c Mice*

| No. | Age in weeks: at start | Age in weeks: at death | Period of injection (weeks) | INH Total dose (mg.) | Lung 1 | Lung 2 | Lung 3 | Lung 4 | Autopsy and histological diagnosis |
|---|---|---|---|---|---|---|---|---|---|
| Males | | | | | | | | | |
| 1150/22 | 5 | 9 | 4 | 20 | | | | | ? Toxaemia; no abnormality found. |
| 1163/51 | 7 | 9 | 2 | 12 | | | | | Lung congested. |
| 1184/86 | 15 | 18 | 3 | 18 | | | | | Bronchopneumonia. |
| 1135/34 | 11 | 25 | 14 | 56 | | | | | Pneumonia. |
| 1150/39 | 5 | 40 | 35 | 96 | + | | | | 2 mm. tumour R.L. at base. Papillary adenoma. |
| 1135/42 | 6 | 43 K | 35 | 78 | | | | | No abnormality found. Lymphatic engorgement. |
| 1135/35 | 11 | 48 K | 35 | 78 | | | | | No abnormality found. Lymphatic engorgement. |
| 1135/33 | 11 | 48 K | 35 | 78 | | | | | No abnormality found. Lymphatic engorgement. |
| 1163/48 | 7 | 67 K | 35 | 88 | | + | | | 2 mm. tumour in R.L. Multifocal papillary adenoma invading bronchioldes. |
| 1163/49 | 7 | 67 K | 35 | 88 | | + | | | 6 mm. tumour in base. Multifocal papillary adenoma surrounded by normal lung. |
| 1163/50 | 7 | 67 K | 35 | 88 | + | + | | | Multiple tumours R.L. and L. lobes. |
| 1150/24 | 5 | 67 K | 35 | 116 | + | | | | Tumours R.A. and L. lobes at base. |
| 1150/26 | 5 | 67 K | 35 | 116 | + | + | | | Multiple tumours at R. and L. bases. Multifocal papillary adenoma. |
| 1184/83 | 15 | 72 K | 35 | 84 | | | | | No abnormality found. |
| 1184/84 | 15 | 72 K | 35 | 84 | + | | | | Multiple subpleural tumours. |
| 1184/82 | 15 | 72 K | 35 | 84 | + | + | + | + | Multiple tumours. Multifocal papillary adenoma and carcinoma; also diffuse alveolar hyperplasia. |
| 1136/36 | 10 | 73 K | 35 | 112 | + | | | | Tumour L. lobe. |
| 1136/37 | 10 | 73 K | 35 | 112 | + | + | | | Multiple tumours. Multifocal papillary adenoma and carcinoma. |
| 1136/38 | 10 | 73 K | 35 | 112 | | | | | No abnormality found. |
| 1185/78 | 16 | 73 K | 35 | 84 | | | | | ,, ,, ,, |
| 1185/80 | 16 | 73 K | 35 | 84 | | | | | ,, ,, ,, |
| 1185/81 | 16 | 73 K | 35 | 84 | | | | | ,, ,, ,, |
| 1185/82 | 16 | 72 K | 35 | 84 | +* | | | | Tumour R.L. lobe. |
| Females | | | | | | | | | |
| 1137/3 | 7 | 11 | 4 | 20 | | | | | Lung congested. |
| 1142/1 | 10 | 11 | 1 | 6 | | | | | ,, ,, |
| 1142/2 | 10 | 11 | 1 | 6 | | | | | ,, ,, |
| 1142/3 | 10 | 11 | 1 | 6 | | | | | ,, ,, |
| 1142/4 | 10 | 12 | 2 | 12 | | | | | ,, ,, |
| 1186/79 | 11 | 55 K | 34 | 84 | + | | | | Tumour R.L. lobe. Papillary adenoma. |
| 1137/44 | 7 | 67 | 48 | 112 | | | | | Lymphatic engorgement. |
| 1197/73 | 36 | 67 K | 9 | 56 | + | + | | | 2 tumours L. |
| 1137/4 | 6 | 70 K | 38 | 112 | + | + | + | + | Small tumours R. Hydrosalpynx. |
| 1137/2 | 6 | 70 K | 38 | 112 | | | | | No abnormality found. |
| 1164/51 | 8 | 71 K | 37 | 84 | + | | | | Small tumours, all lobes. |
| 1164/49 | 8 | 71 K | 37 | 84 | | | | | No abnormality found. |
| 1179/70 | 36 | 86 K | 9 | 56 | + | | | | Multifocal papillary adenoma; leukaemia; ascites. |
| 1179/76 | 36 | 87 K | 9 | 56 | | | | | Mammary adenocarcinoma L.3; metastasis in lung. |
| 1179/72 | 36 | 92 K | 9 | 56 | + | + | | | Many tumours in lung. Multifocal papillary adenoma. Mammary adenocarcinoma R.2. |
| 1179/74 | 36 | 92 K | 9 | 56 | | | | | Lymphomatosis. |

TABLE III.—*INH Feeding by Gastric Intubation—BALB/c Mice*

| No. | Age in weeks: at start | Age in weeks: at death | Duration (weeks) | INH Total dose (mg.) | Lung 1 | Lung 2 | Lung 3 | Lung 4 | Autopsy and histological diagnosis |
|---|---|---|---|---|---|---|---|---|---|
| Males | | | | | | | | | |
| 1141/10 | 10 | 43 | 33 | 138 | | | | | General lymphomatosis, visceral, skeletal, liver, spleen. |
| 2010/3 | 12 | 47 | 35 | 140 | | | | | Septic bronchitis. |
| 1162/81 | 10 | 47 | 37 | 140 | | | | | Bronchopneumonia. |
| 1162/79 | 10 | 50 K | 40 | 143 | | | | | Multifocal lesions R.A. and R.L Bronchopneumonia. |
| 1162/80 | 9 | 52 | 43 | 143 | | | | | Septic bronchitis and lymphangitis. |
| 1183/5 | 7 | 53 | 42 | 145 | + | | | | Bronchiectasis R.M., R.L., R.A.; ? tumour R.U.; bronchopneumonia. Papillary adenoma. |
| 1162/78 | 7 | 53 K | 44 | 148 | | | | | Bronchopneumonia. Adenosis of bronchi. |
| 1138/1 | 8 | 59 K | 44 | 148 | | | | | Bronchiectasis. |
| 1138/2 | 8 | 59 K | 42 | 148 | | + | | | Tumour R.M. Adenoma. |
| 1138/3 | 8 | 59 K | 44 | 148 | +* | | | | Tumour R.L. base. |
| 1138/6 | 8 | 59 K | 44 | 148 | | | | | Bronchopneumonia. Thymoma. |
| 1138/4 | 8 | 59 K | 44 | 148 | | | | | ,, |
| 1141/7 | 8 | 62 K | 42 | 146 | | | | | Bronchitis R. lobes. |
| 1141/9 | 8 | 62 K | 42 | 146 | | | | | Bronchiectasis. |
| 2010/1 | 12 | 65 K | 40 | 140 | | | | | ,, |
| 2010/2 | 12 | 65 K | 40 | 140 | | | | | ,, |
| Females | | | | | | | | | |
| 1139/9 | 9 | 10 | 1 | 4 | | | | | Early penumonic consolidation. |
| 1161/42 | 10 | 14 | 4 | 16 | | | | | Infarcts in liver. |
| 1139/5 | 8 | 17 K | 9 | 36 | | | | | Nothing abnormal found. |
| 1149/22 | 7 | 19 | 12 | 36 | | | | | ,, ,, ,, |
| 1140/10 | 10 | 44 | 34 | 138 | + | | | | Tumour R.A. and L. Small compact papillary adenoma. |
| 1161/41 | 10 | 53 K | 40 | 143 | | | | | Septic bronchitis and bronchiectasis. |
| 1161/45 | 10 | 53 | 40 | 143 | | | | | Septic bronchitis and bronchiectasis. |
| 1149/26 | 7 | 55 | 43 | 145 | | | | | Bronchitis and bronchopneumonia. |
| 1140/8 | 8 | 56 | 42 | 145 | | | | | Peribronchial lymphangitis. |
| 1161/47 | 8 | 58 K | 40 | 143 | | | | | Unresolved bronchopneumonia. |
| 1161/46 | 9 | 59 K | 40 | 143 | | | | | Bronchiectasis and bronchopneumonia. |
| 1149/24 | 9 | 60 K | 40 | 145 | | | | | Bronchiectasis and bronchopneumonia. |
| 1139/6 | 9 | 60 K | 40 | 145 | | | | | Bronchopneumonia. |
| 1139/7 | 9 | 60 K | 40 | 145 | | | | | ,, |
| 1140/13 | 7 | 61 K | 44 | 148 | | | | | Nothing abnormal found. |

and metabolites were recovered from the urine. The above methods of assay were abandoned, therefore, in favour of daily administration of the drug in drinking water.

*INH in drinking water*

Thirty-six male and 14 female C3Hf and 15 male and 20 female BALB/c mice, aged 6 to 14 weeks, were given 0·1% aqueous INH solution in drinking bottles and consumed on average between 2 and 3 ml. (2 to 3 mg. INH) per mouse per day throughout their lives. All those that died naturally were examined as soon as possible after death, and even in those which showed autolytic changes it was generally possible to recognise pulmonary tumours when present. Sick mice at any age were killed, and all survivors were killed when 2 years of age, and immediately autopsied.

*Results.*—At approximately 2·5 mg. of INH per average 20 g. adult mouse the dosage was considered to be the highest tolerable and 8 C3Hf and 1 BALB/c mice died within 24 hours of the experiment. Thus the effective population of 50 C3Hf and 35 BALB/c was selected for tolerance.

No pulmonary tumours occurred in mice which died below 30 weeks of age and only those which survived beyond this period are considered in assessing the carcinogenic response (Tables IV and VI).

---

### EXPLANATION OF PLATES

FIG. 1.—BALB/c Female 1271/34 INH in drinking water (Table VI). Age 98 weeks. Killed in good condition. Total dose of INH = 628 mg. Multiple tumours in lungs. Van Gieson and elastin. ×120. Section shows alveolar hyperplasia (upper right) and perivascular and parabronchial lymphatic engorgement (upper left). In the normal lung there is little evidence of compression by the lesion. The arrangement of elastin (thin black lines) is normal throughout the hyperplastic lesion.

FIG. 2.—Same mouse as above. Another field from the same section shows desquamative alveolar hyperplasia (upper left) merging into papillary adenoma (centre). The underlying normal lung is slightly compressed by the tumour. Elastin is deficient in the papilloma and the alveolar pattern is distorted.

FIG. 3.—Wistar Rat Female 57/35 INH in drinking water (Table VIII). Age 113 weeks. Killed in good condition. Total dose of INH = 13·0 g. Lungs healthy apart from a 3 mm. subpleural lesion on the posterior aspect of the left lobe at the level of the 8th rib. Picro-Mallory and elastin. ×60. Section shows part of a wedge shaped subpleural area of mixed alveolar hyperplasia and early papillary adenoma, with a small focus of distended alveoli containing unidentified non-birefringent elongated foreign bodies partly phagocytosed (circled). Note abrupt transition from normal to hyperplastic epithelium and preservation of normal alveolar pattern. Slight thickening of the pleura is visible (top right).

FIG. 4.—Same slide as above showing pleura as a dense black diagonal line with hyperplastic alveolar epithelium (left) showing partly defective elastic basement membrane as a thin wavy line (A); to the right of the field dilated pleural lymphatics (L) are invaded by more neoplastic epithelium which is devoid of elastin (B). ×340.

FIG. 5.—Wistar Rat Male 75/4 INH in drinking water (Table IX). Age 61 weeks. Killed for rectal prolapse. Total dose of INH = 3·63 g. Unresolved bronchopneumonia and peribronchial adenosis was present but no neoplastic lesions were found in the lungs. There was a fleshy ulcerated tumour about 1 cm. in diameter surrounding the anal prolapse. H. and E. ×110. Section shows a representative field. A keratinised squamous papilloma shows mitotic activity and a tendency to invade the narrow strand of connective tissue stroma.

FIG. 6.—Wistar Rat Female 76/7 INH in drinking water and food (Table IX). Age 78 weeks. Killed for rectal prolapse and anal papilloma. Total dose of INH = 6·23 g. No gross internal lesions found. Van Gieson and elastin ×55. Section shows keratinised papilloma of anal skin (left) and complete squamous metaplasia of rectal glands which are packed with keratin (right). The submucous lymphatics are greatly distended.

FIG. 7.—Wistar Rat Female 76/8 INH in drinking water and food (Table IX). Age 78 weeks. Killed for large prolapsed tumour of anal region. Total dose of INH = 6·23 g. No gross internal lesions found. Van Gieson and elastin ×55. Section through prolapsed tumour shows some functional glands in which mucus appears black, and squamous metaplasia and neoplasia in adjacent glands (the carcinoma is not illustrated).

FIG. 8.—Wistar Rat Female 76/6 INH in drinking water and food (Table IX). Age 108 weeks. Killed for anal polyp and prolapse. A diaphragmatic hernia containing part of the central lobe of the liver was the only internal lesion found. H. and E. ×110. Section shows part of an oedematous polyp (top) and metaplastic and hyperplastic glands (below) with a few normal rectal glands.

1

2

3

4

5

6

7

8

Peacock and Peacock

TABLE IV.—*INH in Drinking Water—C3Hf Mice*

| No. | Age in weeks at start | Age in weeks at death | Duration (weeks) | INH Total dose (mg.) | Lung 1 | Lung 2 | Lung 3 | Lung 4 | Autopsy and histological diagnosis |
|---|---|---|---|---|---|---|---|---|---|
| Males | | | | | | | | | |
| 1230/33 | 12 | 12 | 1 day | 2 | | | | | Toxaemia. |
| 1230/34 | 11 | 12 | 6 days | 12 | | | | | Intense congestion all organs. |
| 1239/49 | 7 | 12 | 5 | 70 | | | | | Killed in extremis. |
| 1226/20 | 14 | 15 | 3 days | 6 | | | | | Early pneumonia. |
| 1226/18 | 14 | 16 | 10 ,, | 20 | | | | | ,, ,, |
| 1238/45 | 14 | 49 | 35 | 130 | | | | | Nil in lungs; haemorrhagic gastroenteritis. |
| 1240/52 | 7 | 72 | 65 | 500 | | | | | ? Tumour L. lung. Epithelial desquamation only. |
| 1222/1 | 17 | 74 K | 57 | 408 | | | | | No abnormality found. |
| 1222/3 | 17 | 74 K | 57 | 408 | | | | | ,, ,, ,, |
| 1222/5 | 17 | 74 K | 57 | 408 | | + | | | Small 1 mm. tumour central. Papillary adenoma. |
| 1224/13 | 14 | 86 | 72 | 480 | | | + | | Tumour R.L. lobe. Alveolar hyperplasia. |
| 1233/39 | 6 | 94 | 88 | 480 | + | + | | | Whole L. lobe replaced by tumour. Diffuse multifocal papillary adenoma. |
| 1223/6 | 10 | 100 K | 90 | 570 | | + | | | Small tumour R.A. lobe. Central adenocarcinoma invading bronchi and veins. |
| 1238/44 | 14 | 101 | 87 | 620 | + | + | + | + | Lungs congested with pale area L. Congestion, alveolar hyperplasia, papillary adenoma. |
| 1239/47 | 8 | 104 K | 96 | 660 | + | + | + | + | Tumour L. lobe. Multifocal papillary adenoma and alveolar hyperplasia. |
| 1239/48 | 8 | 104 K | 96 | 660 | + | | + | | Multiple tumours. Papillary adenoma and alveolar hyperplasia. |
| 1239/50 | 8 | 104 K | 96 | 660 | | + | | | Tumour R.L. lobe. Papillary adenoma. |
| 1240/51 | 9 | 105 K | 96 | 660 | | + | | | Tumour L. base. Papillary adenoma. |
| 1240/52 | 9 | 105 K | 96 | 660 | | + | | | Multiple tumours L. Multifocal papillary adenoma. |
| 1240/53 | 9 | 105 K | 96 | 660 | | + | | + | Tumour L. lobe. Central papillary adenoma and alveolar hyperplasia. |
| 1273/60 | 14 | 105 K | 96 | 660 | | | + | + | 3 Tumours L. lobe. Alveolar hyperplasia. |
| 1273/61 | 14 | 105 K | 96 | 660 | | + | | | ? Tumour L. lobe. Central papillary adenoma. |
| 1224/12 | 14 | 106 K | 92 | 650 | | +* | | | Tumour R.U. lobe. Trabecular hepatoma. |
| 1238/43 | 14 | 106 K | 92 | 650 | + | | | | Large tumour R. hip. Spindle cell sarcoma; lung metastasis, also papillary adenoma. |
| 1224/11 | 14 | 107 | 92 | 650 | | | | | Lung congested. Trabecular hepatoma. |
| 1238/46 | 14 | 110 K | 95 | 660 | + | | | | Tumours L. lobe. Papillary adenoma and carcinoma. |
| 1223/7 | 10 | 111 K | 100 | 680 | | | + | | Tumour L. lobe. Alveolar hyperplasia; lymphomatosis. |
| 1233/40 | 6 | 112 K | 105 | 690 | + | + | + | + | Tumours R. and L. Multifocal papillary adenoma, alveolar hyperplasia. |
| 1233/41 | 6 | 112 K | 105 | 690 | | | | | ? Tumour L. lobe. Lymphatic engorgement. |

TABLE IV. (*contd*)

| No. | Age in weeks at start | Age in weeks at death | Duration (weeks) | INH Total dose (mg.) | Lung 1 | Lung 2 | Lung 3 | Lung 4 | Autopsy and histological diagnosis |
|---|---|---|---|---|---|---|---|---|---|
| 1233/42 | 6 | 112 K | 105 | 690 | + | + | + | + | ? Tumour R.A. and L. Diffuse multifocal alveolar hyperplasia; papillary adenoma and carcinoma. |
| 1228/25 | 8 | 113 K | 105 | 690 | .. | .. | + | + | Dextrocardia. Tumour L. hilum and R. lobe; diffuse alveolar hyperplasia. |
| 1228/26 | 8 | 113 K | 105 | 690 | + | + | + | + | Multiple tumours in lung. Multifocal papillary adenoma, alveolar hyperplasia, trabecular hepatoma. |
| 1223/8 | 10 | 115 K | 105 | 690 | + | + | .. | .. | Multiple tumours. Papillary adenoma and carcinoma. |
| 1223/9 | 10 | 115 K | 105 | 690 | + | .. | .. | .. | Congested lung. Papillary adenoma; metastatic malignant hepatoma. |
| 1224/10 | 14 | 120 K | 105 | 690 | + | .. | + | .. | Multiple tumours. Papillary adenoma, alveolar hyperplasia; multiple hepatoma. |
| 1226/21 | 11 | 120 K | 105 | 690 | .. | .. | + | .. | Tumours R.U. and R.M. lobes. Alveolar hyperplasia. |
| Females | | | | | | | | | |
| 1232/36 | 8 | 7 | 1 day | 2 | .. | .. | .. | .. | Acute toxaemia. |
| 1232/37 | 8 | 7 | 1 ,, | 2 | .. | .. | .. | .. | ,, ,, |
| 1241/55 | 8 | 7 | 6 ,, | 12 | .. | .. | .. | .. | Cannibalised. |
| 1229/31 | 8 | 7 | 1 ,, | 2 | .. | .. | .. | .. | Acute toxaemia. |
| 1229/32 | 8 | 7 | 1 ,, | 2 | .. | .. | .. | .. | ,, ,, |
| 1227/19 | 8 | 12 | 1 ,, | 2 | .. | .. | .. | .. | ,, ,, |
| 1225/14 | 8 | 12 | 1 ,, | 2 | .. | .. | .. | .. | ,, ,, |
| 1225/15 | 7 | 109 K | 94 | 660 | .. | + | .. | .. | Multiple tumours. Bronchopneumonia; papillary adenoma. |
| 1232/38 | 26 | 109 K | 102 | 670 | .. | .. | .. | .. | No abnormality found. |
| 1272/35 | 11 | 113 K | 103 | 670 | + | .. | .. | .. | Multiple tumours. Papillary adenoma. |
| 1231/35 | 12 | 117 K | 105 | 690 | + | + | .. | .. | Multiple tumours. Papillary adenoma with bony metaplasia; hepatoma. |
| 1225/16 | 15 | 119 K | 105 | 690 | + | + | + | + | Multiple tumours. Multifocal papillary adenoma, carcinoma, and alveolar hyperplasia. |
| 1227/22 | 15 | 120 K | 105 | 690 | .. | + | .. | + | ? Multiple tumours L. Papillary adenoma and alveolar hyperplasia. |
| 1227/23 | 15 | 120 K | 105 | 690 | .. | .. | .. | + | ? Tumour R.L. lobe. |

The tables show the survival of each mouse, the period of administration, the total dose of INH and the neoplastic pulmonary lesions classified by anatomical site and histological type. Neoplastic lesions found in untreated control animals killed at comparable ages are also shown (Tables V and VII).

### *Wistar Rats*

*Group I. Subcutaneous injection of INH*

Nine male and 8 female weanling rats about 4 weeks old received 34 subcutaneous injections of 0·2% INH solution (10 mg. per 100 g. body weight) during

TABLE V.—*Controls—C3Hf Mice.*

| No. | Age in weeks at death | Lung 1 | Lung 2 | Lung 3 | Lung 4 | Autopsy and histological diagnosis |
|---|---|---|---|---|---|---|
| Males | | | | | | |
| 1611/1 | 87 | .. | .. | .. | .. | Pneumonia |
| 1604/1 | 91 K | .. | .. | .. | .. | No gross lesions. |
| 1604/2 | 93 K | .. | .. | .. | .. | Lung congested and has metastatic tumours from liver cell carcinoma. |
| 1603/1 | 100 | .. | .. | .. | .. | Enteritis and liver abscesses (Tyzzer's disease). |
| 1603/2 | 101 | .. | .. | .. | .. | ,, ,, ,, ,, |
| 1593/1 | 103 K | .. | .. | .. | .. | No gross lesions. |
| 1606/1 | 104 K | .. | .. | .. | .. | ,, ,, ,, |
| 1616/1 | 104 K | .. | .. | .. | .. | Cystic liver tumour. Trabecular hepatoma (cystic) and liver cell carcinoma. |
| 1596/1 | 105 K | .. | .. | .. | .. | No gross lesions. |
| 1596/2 | 105 K | .. | .. | .. | .. | Liver tumour L. central lobe. Trabecular hepatoma. |
| 1612/1 | 105 K | .. | .. | .. | .. | Lungs congested. |
| 1612/2 | 105 K | .. | .. | .. | .. | No gross lesions. |
| 1602/1 | 106 K | .. | .. | .. | .. | Liver tumours. Multicentric trabecular hepatoma. |
| 1606/2 | 108 K | .. | .. | .. | .. | No gross lesions. |
| 1603/3 | 109 K | .. | .. | .. | .. | Liver tumours and abscesses (Tyzzer's disease). Trabecular hepatoma. |
| 1603/4 | 109 | .. | .. | .. | .. | Multicentric trabecular hepatoma. |
| 1607/1 | 109 K | .. | .. | .. | .. | Hyperplastic mesenteric lymph nodes. |
| 1604/3 | 111 K | .. | .. | .. | + | One small focus of alveolar hyperplasia R.U. lobe. |
| 1604/4 | 111 K | .. | .. | .. | .. | No gross lesions. |
| 1609/1 | 112 K | + | .. | + | .. | Tumour R.U. lobe. Alveolar hyperplasia and papillary adenoma. |
| 1600/2 | 113 K | .. | .. | .. | .. | Lungs congested. Alveolar desquamation. |
| 1611/2 | 113 K | .. | .. | .. | .. | Abscess in R. lachrymal gland. |
| 1589/1 | 116 K | .. | .. | + | .. | One small subpleural tumour L. lobe. Alveolar hyperplasia. |
| 1614/1 | 118 K | .. | .. | .. | .. | No gross lesions. |
| 1614/2 | 119 K | .. | .. | .. | .. | Multiple liver tumours. Pleomorphic hepatoma and liver cell carcinoma. |
| 1614/3 | 119 K | .. | .. | .. | .. | No gross lesions. |
| Females | | | | | | |
| 1608/1 | 96 K | .. | .. | .. | .. | R. ovarian haemorrhagic cyst. |
| 1579/1 | 98 K | .. | .. | .. | .. | No gross lesions. |
| 1597/2 | 98 K | .. | .. | .. | .. | ,, ,, ,, |
| 1594/1 | 103 K | .. | .. | .. | .. | ,, ,, ,, |
| 1613/7 | 105 K | .. | .. | .. | .. | R. ovarian cyst. |
| 1613/8 | 105 K | .. | .. | .. | .. | L. lobe pale. Inflammatory exudate in lung. |
| 1613/9 | 105 K | .. | .. | .. | + | Small lesion apex of R.U. lobe. Alveolar hyperplasia associated with lymphatic engorgement. |
| 1613/10 | 105 K | .. | .. | .. | .. | Congested lung. |
| 1595/1 | 105 K | .. | .. | + | .. | One tumour R.U. lobe. Subpleural alveolar hyperplasia. |
| 1595/2 | 105 K | .. | .. | .. | .. | No gross lesions. |
| 1592/1 | 105 K | .. | .. | .. | .. | L. leg paralysed. Osteogenic sarcoma of lumbar vertebrae. |
| 1627/1 | 108 | .. | .. | .. | .. | No gross lesions. |
| 1605/1 | 110 K | .. | .. | .. | .. | Pyometra. |
| 1605/2 | 110 K | .. | .. | .. | .. | No gross lesions. |
| 1591/1 | 110 K | .. | .. | .. | .. | Endometrial hyperplasia. |
| 1615/1 | 110 K | .. | .. | .. | .. | Uterus enlarged. Recurrent haemorrhages from haemangioma. |
| 1615/2 | 110 K | .. | .. | .. | .. | Large haemangioma of uterine wall. |
| 1615/3 | 110 K | .. | .. | .. | .. | Muscular hypertrophy of uterine wall. |
| 1590/1 | 111 K | + | .. | .. | .. | Septic bronchitis. Papillary adenoma, endometriosis. |
| 1590/2 | 111 K | .. | .. | .. | .. | No gross lesions. |
| 1598/1 | 112 K | .. | .. | .. | .. | Simple ovarian cysts. |
| 1598/2 | 112 K | .. | .. | .. | .. | Small cyst L. ovary. |
| 1601/3 | 112 K | .. | .. | .. | .. | No gross lesions. |
| 1610/3 | 113 K | .. | .. | .. | .. | Small lesions R.U. lobe. Subpleural lymphangitis. Fibromyoma of uterus. |
| 1599/1 | 114 K | .. | .. | .. | .. | Small lesion R.L. lobe. Not confirmed histologically. |
| 1599/2 | 114 K | .. | .. | .. | .. | No gross lesions. |
| 1627/3 | 119 | .. | .. | .. | .. | Obstruction to rectum by pressure of uterine adenocarcinoma ; metastases in lung and pancreas. |
| 1627/4 | 128 | .. | .. | .. | .. | No gross lesions. |

### TABLE VI.—*INH in Drinking Water—BALB/c Mice*

| No. | Age in weeks at start | Age in weeks at death | Duration (weeks) | INH Total dose (mg.) | Lung 1 | Lung 2 | Lung 3 | Lung 4 | Autopsy and histological diagnosis |
|---|---|---|---|---|---|---|---|---|---|
| Males | | | | | | | | | |
| 1250/26 | 10½ | 11 | 3 days | 6 | .. | .. | .. | .. | Acute toxaemia. |
| 1250/28 | 10½ | 11 | 3 ,, | 6 | .. | .. | .. | .. | ,, ,, |
| 1245/7 | 13 | 16 | 3 | 12 | .. | .. | .. | .. | Cannibalised. |
| 1245/8 | 13 | 19 | 6 | 24 | .. | .. | .. | .. | Autolytic. |
| 1247/15 | 14 | 48 | 34 | 126 | .. | .. | .. | .. | Generalised lymphomatosis. |
| 1247/17 | 14 | 72 | 58 | 409 | .. | .. | .. | .. | Pulmonary congestion and oedema. |
| 1271/32 | 12 | 80 K | 72 | 503 | .. | + | .. | .. | Pneumonia. Splenomegaly. |
| 1245/10 | 14 | 81 | 67 | 503 | .. | .. | .. | .. | Head eaten ; lung congested. |
| 1245/9 | 13 | 90 K | 77 | 540 | .. | .. | .. | .. | Visceral lymphomatosis ; lymphatic leukaemia. |
| 1271/31 | 9 | 98 K | 89 | 628 | + | + | .. | .. | Three separate tumours, R. lung. |
| 1271/33 | 9 | 98 K | 89 | 628 | + | .. | .. | .. | Tumour, R.M. lobe. |
| 1271/34 | 9 | 98 K | 89 | 628 | .. | + | .. | .. | Multiple tumours. |
| 1250/27 | 11 | 105 K | 94 | 650 | + | .. | + | .. | Subpleural tumours. |
| 1247/18 | 13 | 106 | 93 | 650 | .. | .. | .. | + | Multiple tumours. Multicentric alveolar hyperplasia. |
| 1247/16 | 14 | 108 K | 94 | 663 | + | + | .. | .. | Multiple lung tumours. Papillary adenomata. Forestomach —hyperkeratosis. |
| Females | | | | | | | | | |
| 1248/20 | 10 | 13 | 3 | 21 | .. | .. | .. | .. | Pneumonia. |
| 1248/22 | 10 | 14 | 4 | 28 | .. | .. | .. | .. | Cannibalised. |
| 1244/5 | 14 | 42 | 28 | 196 | .. | + | .. | .. | Two tumours L. lobe. Adenomata. |
| 1249/25 | 10 | 79 | 69 | 486 | .. | .. | .. | .. | ? Tumour R.L. lobe. Pneumonia. |
| 1246/12 | 10 | 80 | 70 | 492 | .. | .. | .. | + | Tumour R.M. lobe. Haemorrhagic gastroenteritis. |
| 1272/35 | 9 | 98 K | 89 | 628 | + | + | .. | .. | Multiple tumours in lungs. Bony metaplasia in adenomata. |
| 1272/36 | 9 | 98 K | 89 | 628 | .. | .. | .. | .. | No abnormalities found. |
| 1272/37 | 9 | 98 K | 89 | 628 | + | + | + | + | Multiple tumours. Adenoma and hyperplasia. |
| 1272/38 | 9 | 98 K | 89 | 628 | .. | + | .. | + | Multiple tumours. Adenoma and hyperplasia. |
| 1240/13 | 10 | 102 | 92 | 646 | + | .. | .. | .. | Tumours R.U. and R.L. ; splenomegaly. Lymphatic leukaemia. |
| 1248/19 | 11 | 103 | 92 | 648 | + | + | .. | .. | Tumours R.U. ; haemorrhagic gastroenteritis. |
| 1246/11 | 11 | 105 K | 94 | 663 | .. | + | .. | .. | Large multifocal papillary adenoma R.U. |
| 1246/14 | 11 | 105 K | 94 | 663 | + | .. | .. | .. | Tumours R.U. and R.L. Multifocal papillary adenoma. |
| 1248/21 | 11 | 105 K | 94 | 663 | + | + | + | + | Multiple tumours. |
| 1249/23 | 11 | 105 K | 94 | 663 | .. | + | .. | + | Tumours in all lobes. |
| 1249/24 | 11 | 105 K | 94 | 663 | .. | + | .. | + | ,, ,, ,, ,, |
| 1251/29 | 11 | 105 K | 94 | 663 | .. | + | .. | + | Tumours upper left lobe. |
| 1251/30 | 11 | 105 K | 94 | 663 | + | .. | .. | .. | Tumour R.M. Papillary adenoma ; lymphangitis ; L. ovarian cyst. |
| 1244/6 | 14 | 108 K | 94 | 663 | .. | .. | + | .. | Tumours R.U. and L. Alveolar hyperplasia. |
| 1244/4 | 14 | 108 K | 94 | 662 | + | +* | .. | .. | Tumours R.U., R.M., R.A., and L. Adenomata. |

TABLE VII.—*Controls—BALB/c Mice.*

| No. | Age in weeks at death | Lung 1 | Lung 2 | Lung 3 | Lung 4 | Autopsy and histological diagnosis |
|---|---|---|---|---|---|---|
| Males | | | | | | |
| 1584/2 | 96 | + | .. | .. | .. | Lung tumours. Papillary adenoma ; lymphatic engorgement. |
| 1565/1 | 98 K | + | + | .. | .. | Lung tumours. Cystic lymphangectasis, skeletal lymphomatosis. |
| 1178/1 | 99 K | + | .. | + | .. | Lung tumours. Lymphomatous nodules in spleen. |
| 1178/2 | 99 K | .. | .. | .. | .. | Subpleural lymphoid hyperplasia. |
| 1178/3 | 99 K | + | + | .. | .. | Lung tumours. Multifocal papillary adenomata ; carcinoma of liver. |
| 1575/1 | 101 | .. | + | .. | .. | Lung tumours. Multifocal papillary adenomata. |
| 1575/2 | 101 K | .. | .. | .. | .. | No abnormality found. |
| 1575/3 | 101 K | .. | + | .. | + | Lung tumours. Multifocal papillary adenoma and carcinoma of lung. |
| 1572/1 | 101 K | .. | + | .. | .. | Lung tumours. Myeloid leukaemia with liver and spleen enlarged. |
| Females | | | | | | |
| 1583/1 | 83 | + | + | .. | .. | Large tumours in R.U. lobe. Multifocal papillary adenomata. |
| 1577/1 | 101 K | + | .. | .. | .. | Tumour R.U. lobe ; cystic ovary. Papillary adenoma. |
| 1577/2 | 101 K | + | + | + | + | Multiple lung tumours. Alveolar hyperplasia and papillary adenoma. |
| 1577/3 | 101 K | .. | .. | .. | .. | No abnormality in lungs ; cystic R. uterine horn. Endometriosis. |
| 1576/1 | 101 K | .. | .. | .. | .. | No abnormality in lungs ; L.3 mammary adenocarcinoma; visceral lymphomatosis. |
| 1576/2 | 101 K | .. | .. | .. | .. | Large mediastinal nodes ; uterus distended. Pyometra. |
| 1571/3 | 102 K | .. | .. | .. | .. | No abnormality in lungs. |
| 1571/4 | 102 K | + | + | + | + | Multiple tumours. Papillary adenoma, carcinoma and lymphatic leukaemia. |
| 1571/5 | 102 K | +* | .. | .. | .. | Tumour R.L. base. |
| 1573/1 | 102 K | + | .. | .. | + | Multiple lung tumours. Papillary adenoma and alveolar hyperplasia. R.1 and 3 mammary adenocarcinoma ; lymphomatosis. |
| 1574/1 | 102 K | + | .. | + | .. | Large tumour R.L. Multifocal papillary adenoma. |
| 1564/2 | 102 K | + | + | .. | .. | R.L. apical lesion. Papillary adenoma. Visceral lymph nodes enlarged. Inflammatory hyperplasia. |
| 1564/3 | 102 K | .. | .. | .. | .. | Generalised lymphomatosis. |
| 1579/2 | 103 | .. | .. | .. | .. | Lymphomatosis. |
| 1570/3 | 103 K | .. | .. | + | .. | Large nodular spleen ; small nodule R. uterine horn ; lymphomatosis. Alveolar hyperplasia. |
| 1570/4 | 103 K | .. | .. | .. | .. | Tumour in neck and all lymphoid sites. Lymphatic leukaemia. |
| 1563/1 | 103 K | .. | +* | .. | .. | Tumour R.M. ; haemorrhagic ascites. Malignant haemangio-endothelioma. |
| 1563/3 | 104 K | + | .. | + | .. | Small tumour R.U. lobe. Papillary adenoma ; alveolar hyperplasia. |
| 1566/4 | 104 K | .. | + | + | .. | Large tumour R.U. lobe. Papillary adenoma ; alveolar hyperplasia. |
| 1566/5 | 104 K | + | .. | + | .. | Tumour R.A. lobe. Papillary adenoma ; alveolar hyperplasia. |
| 1566/6 | 104 K | + | + | + | + | Multiple tumours in lung. Multifocal papillary adenoma and carcinoma ; lymphomatosis. |
| 1569/1 | 104 K | + | + | .. | .. | Large clear cyst R.U. lobe. Multifocal papillary adenoma ; Uterine mucocele. |
| 1569/2 | 104 K | .. | .. | .. | .. | No abnormality found. |
| 1568/1 | 104 K | + | .. | .. | .. | Tumours R.U. and L. lobes. Multifocal papillary adenoma. |
| 5/1 | 105 K | + | + | .. | .. | Tumour R.M. lobe. Papillary adenoma and carcinoma. R.3 mammary adenocarcinoma. |
| 1567/1 | 105 K | .. | .. | .. | .. | No abnormality found. |
| 1581/1 | 105 K | + | + | .. | .. | Tumour R.L. Multifocal papillary adenoma and carcinoma. |

TABLE VII. (*contd*)

| No. | Age in weeks at death | Lung 1 | Lung 2 | Lung 3 | Lung 4 | Autopsy and histological diagnosis |
|---|---|---|---|---|---|---|
| 1580/1 | 106 K | .. | .. | .. | .. | Huge lymphomata invading kidney and retroperitoneal tissues. |
| 1580/2 | 106 K | + | .. | + | .. | Tumours R.U. and R.L. lobes. Papillary adenoma and alveolar hyperplasia. |
| 1580/3 | 106 K | + | + | + | .. | Large tumours R.U. and R.L. lobes. Papillary adenoma. |
| 1580/4 | 106 K | .. | .. | .. | .. | ? Tumour L. lobe (leukaemic); general lymphomatosis. Lymphatic leukaemia. |
| 1580/5 | 106 K | .. | .. | .. | .. | No abnormality found. |
| 1579/3 | 108 | .. | .. | .. | .. | ,, ,, ,, in lung. Tyzzer's disease of liver. |
| 1579/1 | 109 K | .. | .. | .. | .. | General lymphomatosis. |
| 1579/4 | 109 K | .. | .. | .. | .. | Huge tumour L. lung. Multifocal pleomorphic adenocarcinoma. |
| 1579/2 | 110 | .. | .. | .. | .. | General lymphomatosis. |

a period of 24 weeks, equivalent to a total dose of 680 mg. All the males were killed aged 28 to 29 weeks. Two females were found dead aged 21 weeks, and 6 were killed aged 29 weeks.

*Group II. Intraperitoneal injection of INH*

One male and 6 females 24 weeks old received 20 intraperitoneal injections of 0·2% INH solution over a period of 16 weeks, giving a maximum total dose of 550 mg. Two females were found dead aged 85 and 106 weeks, respectively, and the 1 male and 4 females were killed aged 77, 85, 95 (2) and 106 weeks.

*Results.*—No tumours were found in the lungs or in any other organ or tissue in the animals in Group I and II.

*Group III*

A small group of 4 male and 4 female rats aged 8–10 weeks were given 0·25% INH solution daily in drinking bottles throughout the experiment. A 200 g. rat consumed an average daily dose of 18 mg. INH (Table VIII).

TABLE VIII.—*INH in Drinking Water—Wistar Rats.*

| No. | Age in weeks at start | Age in weeks at death | Duration (weeks) | INH Total dose (gm.) | Lung 1 | Lung 2 | Lung 3 | Lung 4 | Autopsy and histological diagnosis |
|---|---|---|---|---|---|---|---|---|---|
| Males | | | | | | | | | |
| 60/42 | 6 | 17 K | 11 | 1·8 | .. | .. | .. | .. | Bronchiectasis with squamous metaplasia. |
| 58/38 | 6 | 30 K | 24 | 2·7 | .. | .. | .. | .. | Pulmonary interstitial hymphangitis. |
| 58/37 | 7 | 40 K | 33 | 4·0 | .. | .. | .. | .. | Paraplegic; cause not found. |
| 60/41 | 6 | 48 K | 42 | 5·0 | .. | .. | .. | .. | Congestion and bronchitis with local necrosis. |
| Females | | | | | | | | | |
| 57/36 | 7 | 40 | 33 | 4·0 | .. | .. | .. | .. | Congestion and local necroses in bronchi. |
| 59/39 | 6 | 54 K | 48 | 5·8 | .. | .. | .. | .. | Bronchopneumonia. |
| 59/40 | 7 | 87 K | 80 | 11·0 | .. | .. | .. | .. | Pneumonia and bronchiectasis. |
| 57/35 | 7 | 113 K | 106 | 13·0 | + | .. | + | + | Foreign bodies in peripheral alveolus; local lymphangitis, Alveolar hyperplasia and papillary adenoma (Fig. 3 and 4). |

*Results*.—There was a high incidence of respiratory lesions; bronchitis, bronchiectasis and broncho-pneumonia were present at death in all but the youngest, but only the last surviving female aged 113 weeks showed central and subpleural alveolar hyperplasia and papillary adenoma (Fig. 3 and 4) comparable with the lesions so described in mice (Peacock and Peacock, 1966).

*Group IV*

A group of 14 males and 9 females were given INH in drinking water and food for 20 weeks by which time they showed loss of appetite and consequent loss of weight because they drank less than controls. Those that died were found to be constipated, some with the whole length of the colon distended with faeces. The INH solution was therefore withheld for a week and replaced by tap water which the rats drank avidly. Thereafter the INH solution was given on alternate weeks only, for a further 35 weeks by which time the animals had regained and maintained normal weight. However, they did not drink as much as controls and the drug was therefore mixed with the food in quantity to give the same average daily dose supplemented by tap water *ad libitum.*

The animals tolerated this regime well and the survivors were killed when approximately 2 years old.

*Results*.—The course of the experiment is shown in Table IX. Only one hyperplastic and neoplastic subpleural pulmonary lesion was found. Many animals had chronic respiratory infections but there was an unexpected incidence of 5 cases of rectal prolapse, 4 of which were associated with squamous metaplasia of the glands of the terminal part of the rectum and with multifocal squamous papilloma of the anal margin (Fig. 6, 7, 8), one showing areas of possibly early squamous carcinoma (Fig. 5).

Further reference to these cases will be made under Discussion.

No comparable lesions were seen in 15 male and 14 female Wistar rats kept during the experimental period as controls. (Table X).

### *Desert Rats* (Meriones lybicus)

*INH in drinking water*

Seventeen males and 11 females aged 4 months were given INH daily in drinking water as a 0·25% solution throughout their lives.

*Results*.—The results are shown in Table XI.

No lung tumours were found but in the two oldest males, killed at 128 weeks of age, there were small foci of subpleural lymphatic engorgement of the type associated in mice and rats with alveolar hyperplasia. No tumour at any site in either sex was found in 5 male and 6 female desert rats of comparable ages (all over 1 year).

### *Hamsters* (Mesocricetus auratus)

*Intraperitoneal injection of INH*

Three males and 2 females aged 8 weeks received 15 intraperitoneal injections of 0·1% INH in water, equivalent to 10 mg. INH per 100 g. body weight and a total dose of 90 mg.

*Results*.—One male died aged 51 weeks and 2 were killed at 66 and 67 weeks,

TABLE IX.—*INH in Drinking Water and Food—Wistar Rats.*

| No. | Age in weeks at start | Age in weeks at death | Duration (weeks) | INH Total dose (gm.) | Lung 1 | Lung 2 | Lung 3 | Lung 4 | Autopsy and histological diagnosis |
|---|---|---|---|---|---|---|---|---|---|
| Males | | | | | | | | | |
| 60/42 | 7 | 17 K | 10 | 1·8 | .. | .. | .. | .. | Bronchiectasis and squamous metaplasia. |
| 78/13 | 7 | 18 K | 11 | 1·9 | .. | .. | .. | .. | No abnormality found. |
| 81/18 | 7 | 19 K | 13 | 1·61 | .. | .. | .. | .. | No abnormality found. |
| 81/19 | 7 | 19 K | 13 | 1·61 | .. | .. | .. | .. | No abnormality found. |
| 78/12 | 7 | 21 K | 13 | 2·07 | .. | .. | .. | .. | Subpleural lymphangitis and congestion. |
| 75/4 | 7 | 61 K | 23 | 3·63 | .. | .. | .. | .. | Bronchopneumonia. Bronchial adenosis. Anal papilloma, and early carcinoma (Fig. 5). |
| 64/1 | 7 | 88 K | 49 | 6·39 | .. | .. | .. | .. | Left lung collapsed. |
| 75/5 | 7 | 96 K | 51 | 6·51 | + | .. | + | .. | Bronchiectasis and partial collapse. Alveolar hyperplasia. |
| 79/15 | 7 | 97 | 66 | 6·54 | .. | .. | .. | .. | Lungs normal. Pleomorphic sarcoma R. hip and flank. |
| 79/14 | 7 | 98 | 67 | 6·56 | .. | .. | .. | .. | Lungs congested. |
| 80/17 | 7 | 105 K | 69 | 6·70 | .. | .. | .. | .. | Bronchiectasis and bronchial adenosis. |
| 74/2 | 7 | 109 K | 71 | 6·72 | .. | .. | .. | .. | Bronchiectasis and bronchial adenosis. |
| 74/3 | 7 | 109 K | 72 | 6·72 | .. | .. | .. | .. | Pigmented macrophages in lymphatics. |
| 80/16 | 7 | 109 K | 72 | 6·72 | .. | .. | .. | .. | Bronchiectasis; abscess in pyloric wall. |
| Females | | | | | | | | | |
| 77/9 | 7 | 19 K | 12 | 1·63 | .. | .. | .. | .. | No abnormality found. Persistent rectal prolapse. |
| 76/7 | 7 | 78 K | 53 | 6·23 | .. | .. | .. | .. | Prolapsed rectum. Keratinised papilloma of anal skin. (Fig. 6). |
| 76/8 | 7 | 78 K | 53 | 6·23 | .. | .. | .. | .. | Bronchopneumonia; prolapse. Keratinising papilloma and squamous metaplasia and carcinoma of anal glands. (Fig. 7). |
| 77/11 | 8 | 97 | 66 | 6·54 | .. | .. | .. | .. | Malignant hepatoma; metastases in lung. |
| 77/10 | 8 | 100 K | 67 | 6·54 | .. | .. | .. | .. | Pyometra and subphrenic abscess. |
| 76/6 | 7 | 108 K | 70 | 6·72 | .. | .. | .. | .. | Lung congested. Nodular hyperplasia of liver. Papillomatous anal polyp. (Fig. 8). |
| 82/20 | 7 | 109 K | 71 | 9·3 | .. | .. | .. | .. | Tumour L. side of thyroid. Cystic ovaries. Thyroid carcinoma. |
| 82/21 | 8 | 109 K | 70 | 9·3 | .. | .. | .. | .. | Bronchiectasis. Hepatoma, cystic ovaries. |
| 82/22 | 8 | 109 K | 70 | 9·3 | .. | .. | .. | .. | Bronchiectasis and bronchial adenosis. |

respectively. The females died at 69 and 89 weeks, respectively. No tumours of the lung or other organ were found.

*INH in drinking water*

Seven males and 9 females were given 0·25% INH solution in drinking water daily throughout their lives.

*Results.*—No pulmonary tumours were seen. The course of the experiment and total dosage are shown in Table XII.

TABLE X.—*Controls—Wistar Rats*

| No. | Age in weeks at death | Lung 1 | 2 | 3 | 4 | Autopsy and histological diagnosis |
|---|---|---|---|---|---|---|
| Males | | | | | | |
| 62/5 | 75 | .. | .. | .. | .. | Peribronchial lymphatic engorgement. |
| 65/13 | 90 K | .. | .. | .. | .. | Emphysema and lymphangitis. |
| 61/1 | 91 K | .. | .. | .. | .. | Bronchopneumonia. |
| 69/20 | 100 | .. | .. | .. | .. | Desquamative pneumonitis. |
| 73/1 | 102 K | .. | .. | .. | .. | Enteritis. |
| 71/27 | 104 K | .. | .. | .. | .. | No abnormality found. |
| 62/4 | 106 K | .. | .. | .. | .. | Early bronchiectasis ; abscess in mastoid sinus. |
| 61/3 | 107 K | .. | .. | .. | .. | Peribronchial lymphangitis. Fatty liver. |
| 61/2 | 108 | .. | .. | .. | .. | Bronchiectasis, chronic peritonitis. ? Healed intestinal perforation. |
| 66/14 | 108 K | .. | .. | .. | .. | Bronchiectasis. |
| 72/28 | 110 K | .. | .. | .. | .. | Haemorrhagic infarct of spleen. |
| 71/24 | 110 K | .. | .. | .. | .. | No abnormality found. |
| 65/11 | 110 K | .. | .. | .. | .. | No abnormality found. |
| 65/12 | 110 K | .. | .. | .. | .. | No abnormality found. |
| 71/25 | 112 K | .. | .. | .. | .. | Fatty liver. |
| Females | | | | | | |
| 64/9 | 82 K | .. | .. | .. | .. | No abnormality found. |
| 63/7 | 98 K | .. | .. | .. | .. | Fibroadenoma of breast ; adenocarcinoma of ovary. |
| 64/10 | 98 K | .. | .. | .. | .. | Bronchopneumonia. |
| 66/15 | 102 K | .. | .. | .. | .. | No abnormality found. |
| 68/18 | 105 | .. | .. | .. | .. | Scirrhous adenocarcinoma of breast ; metastases in lung. |
| 67/17 | 106 K | .. | .. | .. | .. | Adenosis of breast. |
| 70/23 | 106 K | .. | .. | .. | .. | Congested lung. Interstitial nephritis. |
| 68/19 | 107 K | .. | .. | .. | .. | Congested lung. |
| 72/29 | 109 K | .. | .. | .. | .. | Myoma of uterus. |
| 67/16 | 109 K | .. | .. | .. | .. | Cystic endometriosis. |
| 70/21 | 111 K | .. | .. | .. | .. | Chronic interstitial nephritis. |
| 70/22 | 111 K | .. | .. | .. | .. | Columnar cell adenocarcinoma of uterus. |
| 63/8 | 113 K | .. | .. | .. | .. | Cystic adenocarcinoma of ovary, invading small intestine. |
| 63/6 | 114 K | .. | .. | .. | .. | Septic bronchitis. Anaplastic ovarian carcinoma invading kidney and muscle ; necrotic metastases in lung. |

### DISCUSSION

Only in C3Hf mice was the incidence of lung tumours clearly increased by treatment with INH (Tables IV and V).

In our short term groups of BALB/c mice given INH by gastric intubation the results were confused by the high incidence of unresolved septic bronchitis, bronchopneumonia and bronchiectasis associated in 2 animals with papillary adenoma.

In long term experiments there was no significant difference between the experimental and control BALB/c mice at about 2 years of age. C3Hf mice, on the other hand, with a lower spontaneous incidence of pulmonary tumours, showed a significantly increased incidence of tumours attributable to INH.

We have adopted a method of classification of lung tumours in mice based on the anatomical site of origin and histological criteria of neoplasia (Peacock and Peacock, 1966) and this method has been used also in examining the lungs of the other species of rodent.

Positive records under each heading were all confirmed, unless otherwise stated, by examination of serial sections through the macroscopically visible lesions. Often smaller lesions involving even a few cells were discovered during such examinations. No attempt has been made to enumerate the lesions of each type in this study because many lesions are obviously multicentric in origin and some are so small or so early that they might be debatable.

Despite these limitations it is obvious that INH is carcinogenic for C3Hf mice, but not obvious in the case of BALB/c mice, in our experiments.

Bianciflori and Ribacchi (1962) showed that daily administration of 2 mg. of INH by gastric intubation caused pulmonary tumours in all of 38 virgin female BALB/c mice which survived for 46 weeks following the start of treatment at 8 weeks of age. Their total dosage was 502 mg. They saw no pulmonary tumours in untreated controls.

In our experiments approximately the same total dosage was given over 80–120 weeks, with similar results in BALB/c mice of both sexes, but at that age the untreated controls also had a high incidence of spontaneous tumours.

The tables show the sex, age and duration of exposure to INH, the total dose per animal and the incidence and site of origin of hyperplastic and neoplastic

TABLE XI.—*INH in Drinking Water*—Meriones lybicus

| No. | Age in weeks at start | Age in weeks at death | Duration (weeks) | INH Total dose (g.) | Autopsy and histological diagnosis |
|---|---|---|---|---|---|
| Males | | | | | |
| 18/26 | 5 | 39 | 34 | 3·52 | Lungs congested. Liver congested with pale spots (fatty change). |
| 18/25 | 5 | 39 | 34 | 3·52 | Vesical calculus obstructing urethra. Small cortical adenoma of adrenal |
| 9/7 | 18 | 56 K | 38 | 4·04 | Killed for foot injury. Subpleural lymphatic engorgement. |
| 9/8 | 18 | 56 K | 38 | 4·04 | Killed for foot injury. Vesical calculus, but not causing obstruction |
| 11/13 | 19 | 88 | 69 | 7·22 | Autolytic. No gross lesions. |
| 9/9 | 18 | 96 | 78 | 8·25 | ,, ,, ,, ,, |
| 8/5 | 18 | 106 | 88 | 9·27 | ,, ,, ,, ,, |
| 16/23 | 18 | 109 | 88 | 9·27 | Pneumonia. |
| 8/4 | 18 | 109 | 91 | 9·57 | Ureteric calculi ; cystitis. |
| 8/6 | 18 | 111 K | 93 | 9·81 | Spastic muscular paralysis. C.N.S. appears normal. |
| 11/14 | 19 | 117 K | 98 | 11·28 | Killed in good condition. No gross lesions found. |
| 10/12 | 19 | 126 | 108 | 11·37 | Lungs congested and slightly oedematous. |
| 17/24 | 18 | 127 | 109 | 11·52 | No gross lesions. |
| 10/10 | 19 | 127 | 108 | 11·38 | Small cysts in liver. Cholangiectasis and fatty change. |
| 10/11 | 19 | 127 | 108 | 11·52 | No gross or histological abnormalities. |
| 12/15 | 19 | 128 K | 109 | 11·43 | Killed in good condition. Slight focal alveolar hyperplasia and prominent subpleural lymphatics. |
| 12/16 | 19 | 128 K | 109 | 11·43 | Killed in good condition. Slight focal alveolar hyperplasia and prominent subpleural lymphatics. |
| Females | | | | | |
| 14/19 | 19 | 60 | 41 | 4·32 | Autolytic. No gross lesions. |
| 14/20 | 19 | 76 | 57 | 5·98 | Accidental hyperthermia. Lungs congested. |
| 19/27 | 5 | 101 | 96 | 10·08 | Teeth caught in cage bars. Autolytic. |
| 19/28 | 5 | 114 K | 109 | 11·43 | Hepatoma with advanced fatty change. |
| 7/1 | 18 | 120 K | 102 | 10·75 | No gross or histological abnormality. |
| 7/2 | 18 | 120 K | 102 | 10·75 | ,, ,, ,, ,, ,, |
| 7/3 | 18 | 120 K | 102 | 10·75 | ,, ,, ,, ,, ,, |
| 15/21 | 19 | 127 K | 108 | 11·38 | ,, ,, ,, ,, ,, |
| 15/22 | 19 | 127 K | 108 | 11·38 | ,, ,, ,, ,, ,, |
| 13/17 | 19 | 128 | 109 | 11·43 | ,, ,, ,, ,, ,, |
| 13/18 | 19 | 128 | 109 | 11·43 | Liver shows fatty change. |

lesions. Where nothing is shown under headings Lung 1 to 4 in the tables, no hyperplastic or neoplastic lesions were present, though in many of the animals there were inflammatory lesions of the lungs. In all cases the lungs were inspected and with a few exceptions routine random histological sections were examined, even in those that appeared normal.

In Wistar rats the only pulmonary lesions which might be attributable to INH were 2 cases of subpleural alveolar hyperplasia and one of early papillary adenoma (Fig. 3 and 4).

The occurrence of one case of persistent rectal prolapse and 4 cases of prolapse associated with squamous metaplasia of the columnar epithelium of the anal canal and varying degrees of further neoplastic progression was remarkable in a group of 22 rats.

On tracing the pedigree of the affected rats it was found that the 4 females were litter mate sisters of a family of 6 sisters and 4 brothers.

The first to develop prolapse of the rectum was killed aged 19 weeks and was found to be constipated but showed no macroscopic lesion of the rectum or anal canal and only the lungs were examined histologically and found to be normal. Of the other 2 sisters one died aged 97 weeks and was found to have multifocal haemorrhagic hepatomata in the lower right lobe and central lobes of the liver with metastases in the lung. All showed the structure of hepatocellular carcinoma. The remaining sister was killed aged 100 weeks. The right horn of the uterus was the seat of pyometra and there were enlarged para-aortic lymph nodes and there was a large hard mass in the upper abdomen adherent to the liver and to the abdominal wall which proved to be an abscess. The other 3 sisters with

TABLE XII.—*INH in Drinking Water—Hamsters.*

| No. | Age in weeks at start | Age in weeks at death | Duration (weeks) | INH Total dose (g.) | Autopsy and histological diagnosis |
|---|---|---|---|---|---|
| Males | | | | | |
| 25/13 | 8 | 31 | 23 | 1·98 | No gross lesions. |
| 22/7 | 8 | 77 K | 69 | 6·0 | Killed in poor condition. No gross lesions. |
| 19/25 | 48 | 81 K | 33 | 2·91 | Killed in poor condition. Nephritis; casts in tubules. |
| 26/17 | 21 | 82 K | 61 | 5·36 | Killed in poor condition. Chronic nephritis; hyaline casts in tubules. |
| 26/18 | 21 | 82 K | 61 | 5·36 | Killed in good condition. No lesions found. |
| 25/14 | 7 | 97 | 90 | 7·88 | Lobar pneumonia. Cholangiectasis. |
| 25/25 | 7 | 97 | 90 | 7·88 | Lungs congested. |
| Females | | | | | |
| 24/12 | 9 | 26 | 17 | 1·47 | Partly cannibalised. Lungs pneumonic. |
| 19/28 | 7 | 40 | 33 | 2·90 | Caught in cage. Stomach and intestine empty. No lesions found. |
| 20/1 | 30 | 45 K | 15 | 1·33 | Killed in poor condition. Lungs congested. |
| 21/6 | 19 | 48 | 29 | 2·58 | Lungs congested. |
| 20/2 | 38 | 54 K | 16 | 1·41 | Killed in poor condition. No gross lesions found. |
| 23/9 | 10 | 59 | 49 | 4·33 | Partly cannibalised. Lungs congested. |
| 23/10 | 8 | 77 K | 69 | 6·0 | Killed in poor condition. Hepatoma; squamous papilloma, in forestomach. |
| 24/14 | 7 | 78 K | 71 | 6·23 | Killed in poor condition. Liver—focal haemangioma. |
| 24/11 | 56 | 89 K | 33 | 2·91 | Prolapsed rectum; no lesions found. |

prolapse and anal tumours were caged together throughout their lives. Two were killed aged 78 weeks for prolapse and perineal tumours, one had a keratinised multifocal papilloma apparently originating in the anal skin (Fig. 6) ; the other had a larger keratinising squamous papilloma and early carcinoma (not illustrated) associated with squamous metaplasia of the columnar cell glands of the prolapsed rectum and anal canal (Fig. 7). The third sister was killed aged 108 weeks in good condition and was found to have a small wart in the anal canal ; histologically this was a keratinising squamous papilloma. There was a diaphragmatic hernia of part of the central lobe of the liver which showed no histological abnormality ; otherwise no abnormality was found. No constipation was present in these 3 sisters at the time of death. The one male which was killed aged 61 weeks for prolapse had a squamous papilloma and early carcinoma of the anal canal (Fig. 5) and also had unresolved bronchopneumonia and adenosis of the bronchial epithelium and an abscess in the right maxilla.

Its cage mate, killed at 96 weeks, also had bronchiectasis and a small patch of alveolar hyperplasia.

The male with anal papilloma was half-brother to the 4 sisters with similar lesions, their mothers were litter mate sisters both mated to the same litter mate brother. However, the grandparents were of heterozygous Wistar stock.

The complexity of the aetiological factors in these rats is apparent. All were exposed to daily dosage of INH, all were infested with *Demodex folliculorum*, and they were closely related. Constipation, a factor predisposing to prolapse, was common to most of the rats on INH when it was given in drinking water but not in the latter part of the experimental period when it was mixed with the food, and it is interesting to note the absence of macroscopic tumours in the first of the sisters killed for prolapse at 14 weeks of age. Unfortunately no sections were taken in this case. The presence of *Demodex* infestation of the perineal pilosebaceous glands in these 5 rats is recorded without prejudice. Unfortunately the observation was made after the death of all the experimental and control animals during histological study and consequently no contemporary control observations are available. However, one untreated female survivor from the same stock of Wistar rats was killed in good condition at 75 weeks of age and immediately examined. No lesions were found in any organ. Serial sections of skin from perineal and other sites showed general infestation of pilosebaceous follicles by *Demodex folliculorum*, but no inflammatory or other tissue reaction. It seems probable, therefore, that the whole stock of Wistar rats was infested though they showed no signs of dermatitis. Desert rats and hamsters showed no lesions attributable to INH. The ears of the desert rats are frequently infested by *Demodex folliculorum* where it does not seem to cause irritation or gross or microscopic lesions, but some years ago we observed one case of spontaneous squamous carcinoma of the external auditory meatus associated with *Demodex* infestation in this species. We have never found *Demodex* infestation in hamsters or mice.

Symptomless infestation by *Demodex folliculorum* of the pilosebaceous follicles of facial and mammary regions occurs with undetermined frequency in man and is usually demonstrable in our experience in the neighbourhood of rodent ulcers.

Though there is insufficient evidence to justify any opinion on a possible aetiological role of the *Demodex* in skin cancer, it seems desirable to record its presence when observed.

### CONCLUSIONS

While our primary concern was to assess the effect of INH on the lungs of rodents, the possibility of induced lesions in other tissues had to be borne in mind.

Our results emphasise the peculiar susceptibility of mice to alveolar hyperplasia and papillary adenoma of the lung. The results in Wistar rats are not easy to interpret and call for further investigation, but as regards lesions in the lungs, the evidence for carcinogenicity of INH is slight. It is suggested that INH may have promoted progression from squamous metaplasia to neoplasia in the epithelium of the lower rectum rendered metaplastic by prolapse caused in turn by constipation. INH may also have acted directly on the squamous epithelium of the anal margin to induce keratinising papilloma and carcinoma. The absence of demonstrable neoplastic response in two of the four species of rodent tested under conditions thought to be realistic in terms of human dosage with the same drug encourages us to suggest that INH at the dosage level used in human cases of tuberculosis represents an acceptable carcinogenic hazard.

It is another matter to advocate its use as a prophylactic in healthy babies. While there seems to be no way of predicting whether or not INH should be regarded as more or less potentially carcinogenic for man than for rodents, it would be prudent to assume that there is some degree of risk and to use it only as a curative drug and limit as far as possible the duration of its exhibition in each case.

### SUMMARY

Isoniazid given in drinking water proved toxic for mice, Wistar rats, desert rats and hamsters at daily dosage of more than 10 mg. per 100 g. body weight. The highest tolerable daily dosage levels were established for each strain or species of rodent, and were reduced temporarily if toxic effects developed.

The methods of gastric intubation, subcutaneous or intraperitoneal injection for administering INH were abandoned in favour of oral administration in drinking water or food.

Hyperplastic and neoplastic lesions originating in the pulmonary alveoli were induced by INH given in drinking water at between 2 and 3 mg. daily in C3Hf mice killed after approximately 2 years' treatment. Similar dosage in BALB/c mice gave equivocal results because of the high spontaneous incidence of similar lesions in untreated controls at 2 years of age.

Wistar rats given INH daily in drinking water lost appetite and weight and were constipated. Five out of 22 developed rectal prolapse and 4 of these had squamous metaplasia and papilloma and two early squamous carcinoma of the anal margin. Of these 5 rats 4 were litter mate sisters and the fifth was a half brother. The possible aetiological factors are discussed.

Wistar rats showed very little evidence of pulmonary response to INH—2 cases of local subpleural alveolar hyperplasia and early adenoma out of 22 at risk.

Desert rats and hamsters showed no neoplastic response to INH at similar dosage levels for 2 years.

It is concluded that INH is an acceptable risk when used as a curative drug for tuberculosis in man, but that it should not be used prophylactically in healthy babies.

### REFERENCES

BIANCIFIORI, C. AND RIBACCHI, R.—(1962) *Nature, Lond.*, **194**, 488.

CHALMERS, J. G.—(1965) *Br. J. Cancer*, **19**, 430.

JUHASZ, J., BALÒ, J. AND KENDREY, G.—(1957) *Z. Krebsforsch.*, **62**, 188.

PEACOCK, P. M. AND PEACOCK, P. R.—(1966) *Br. J. Cancer*, **20**, 127.


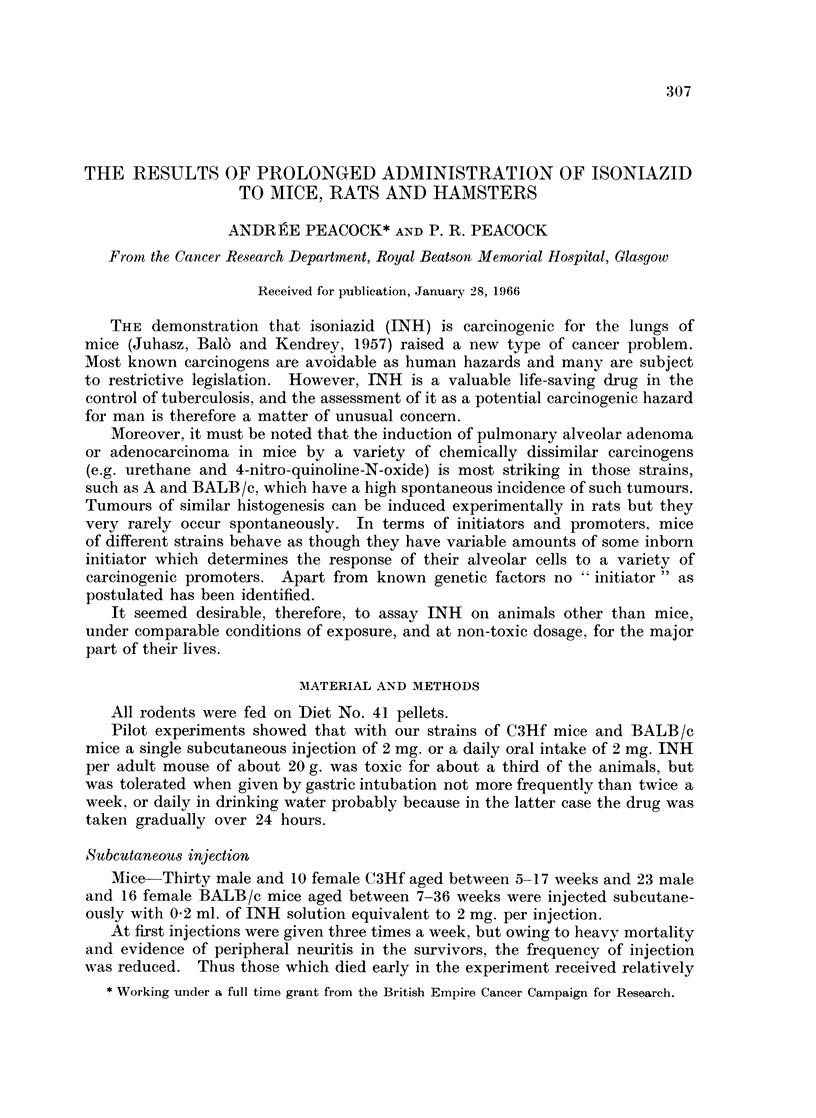

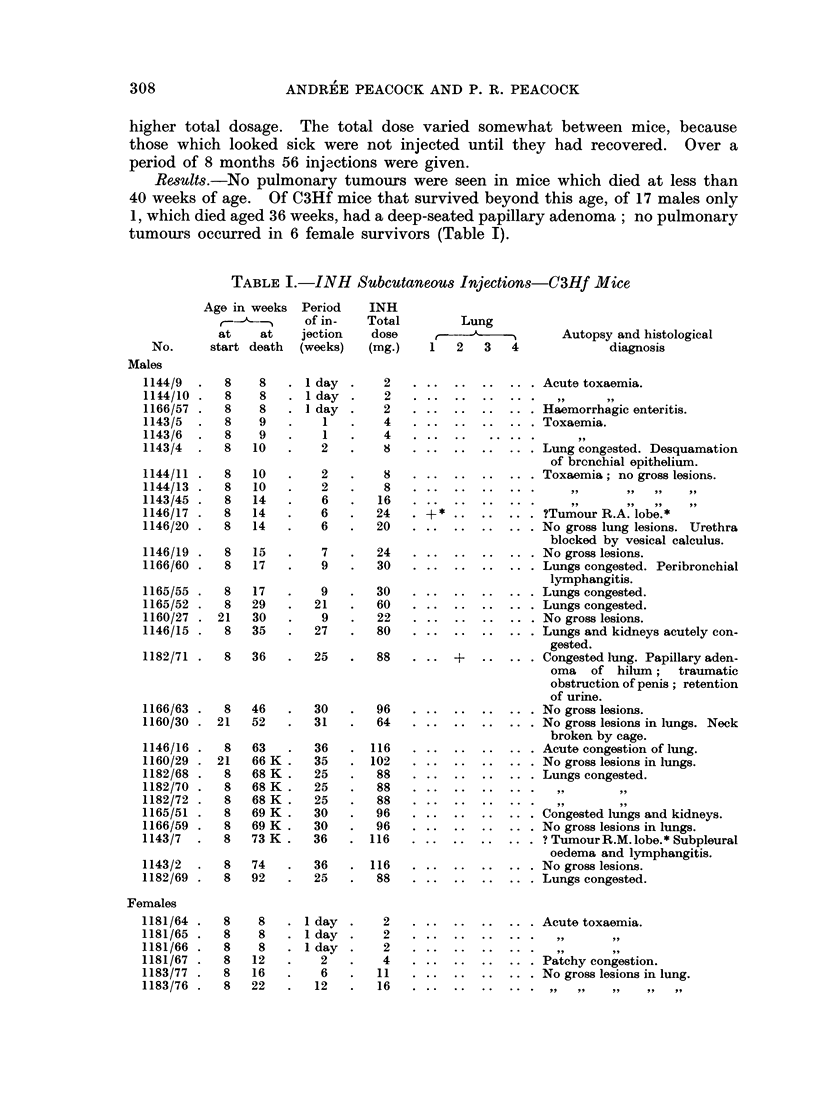

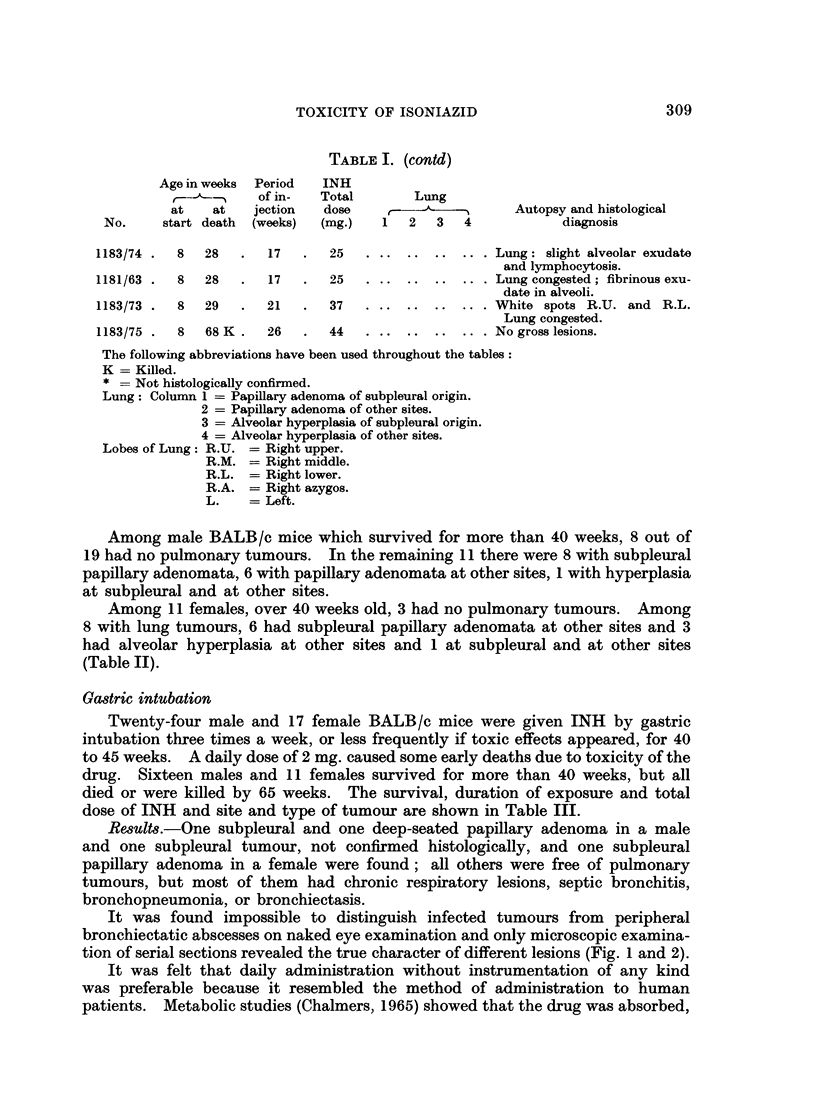

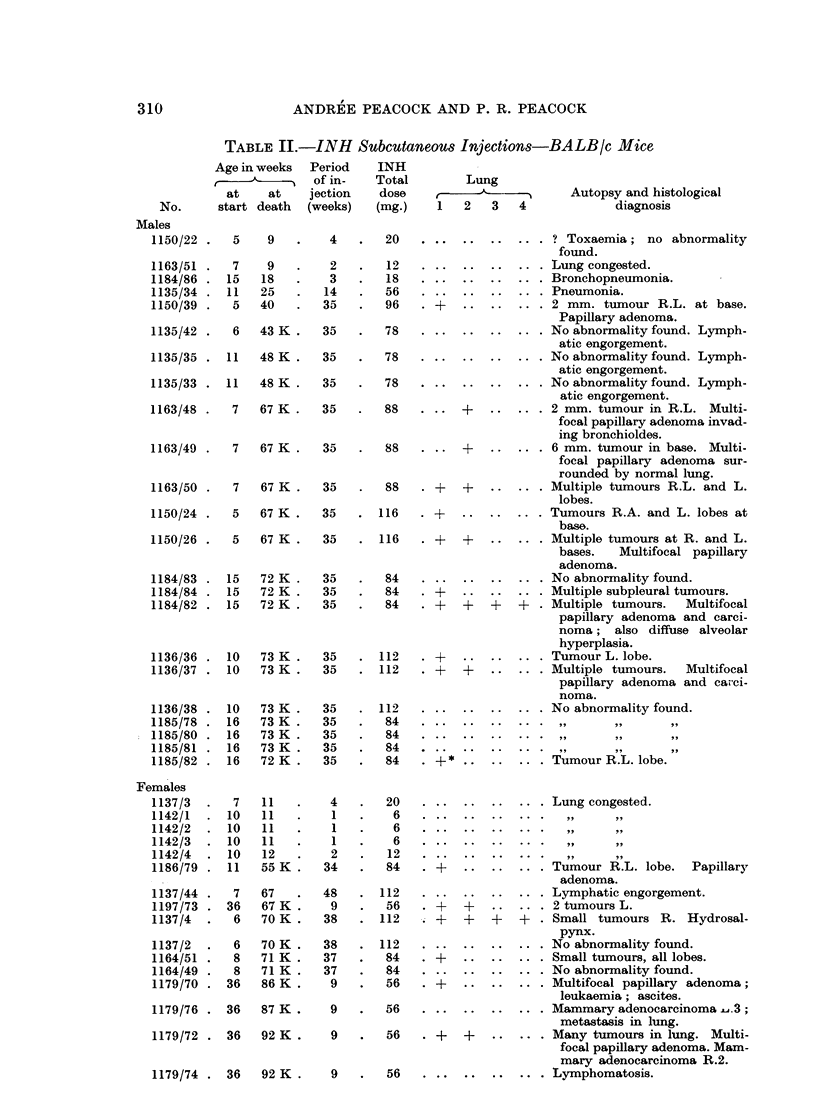

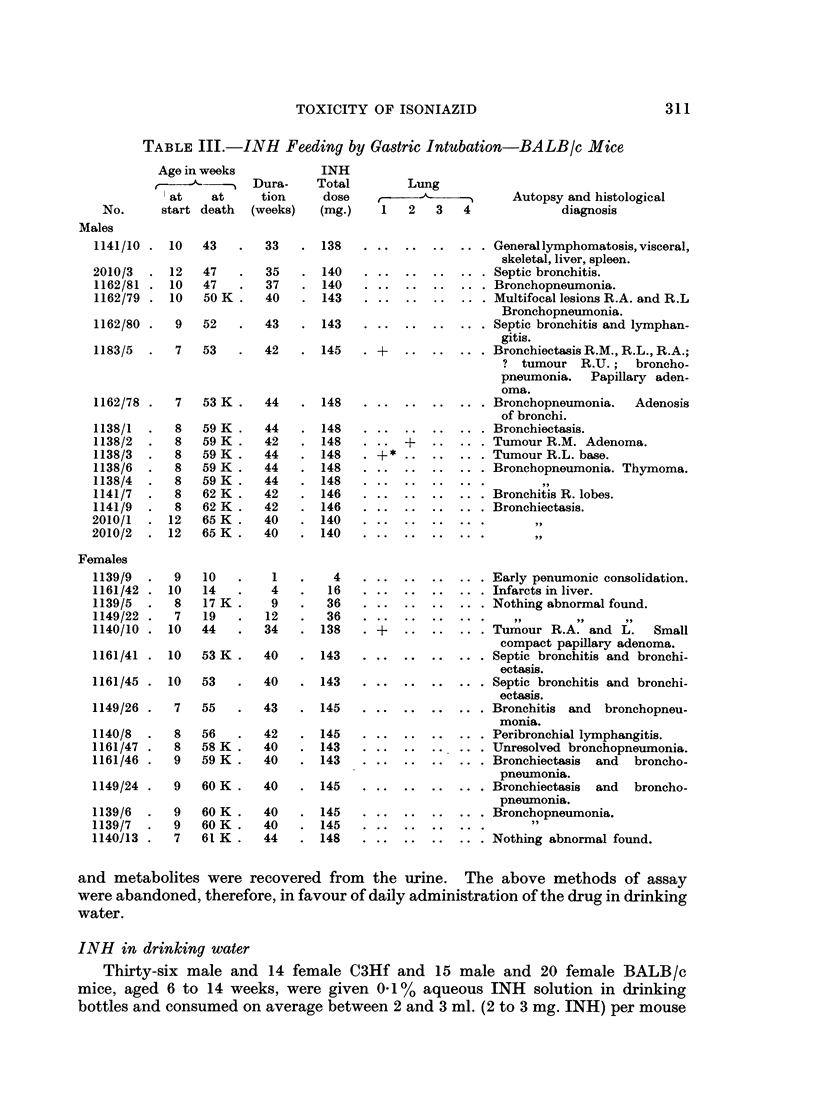

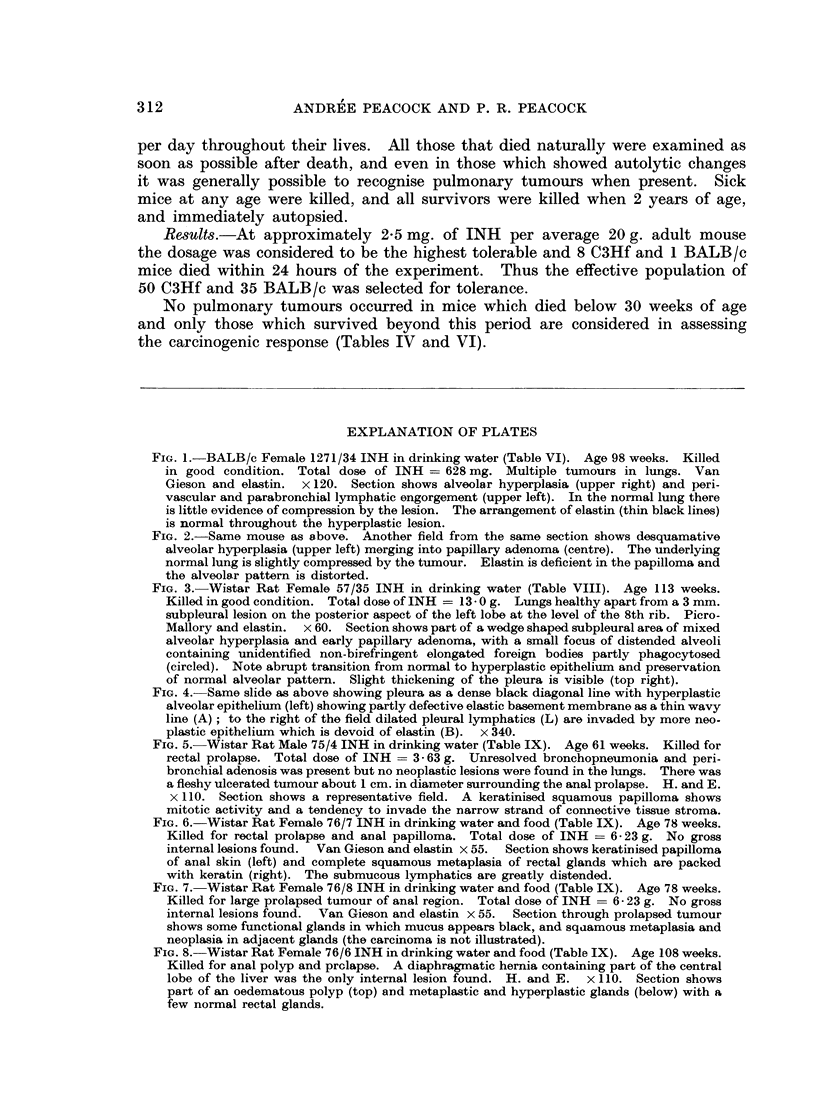

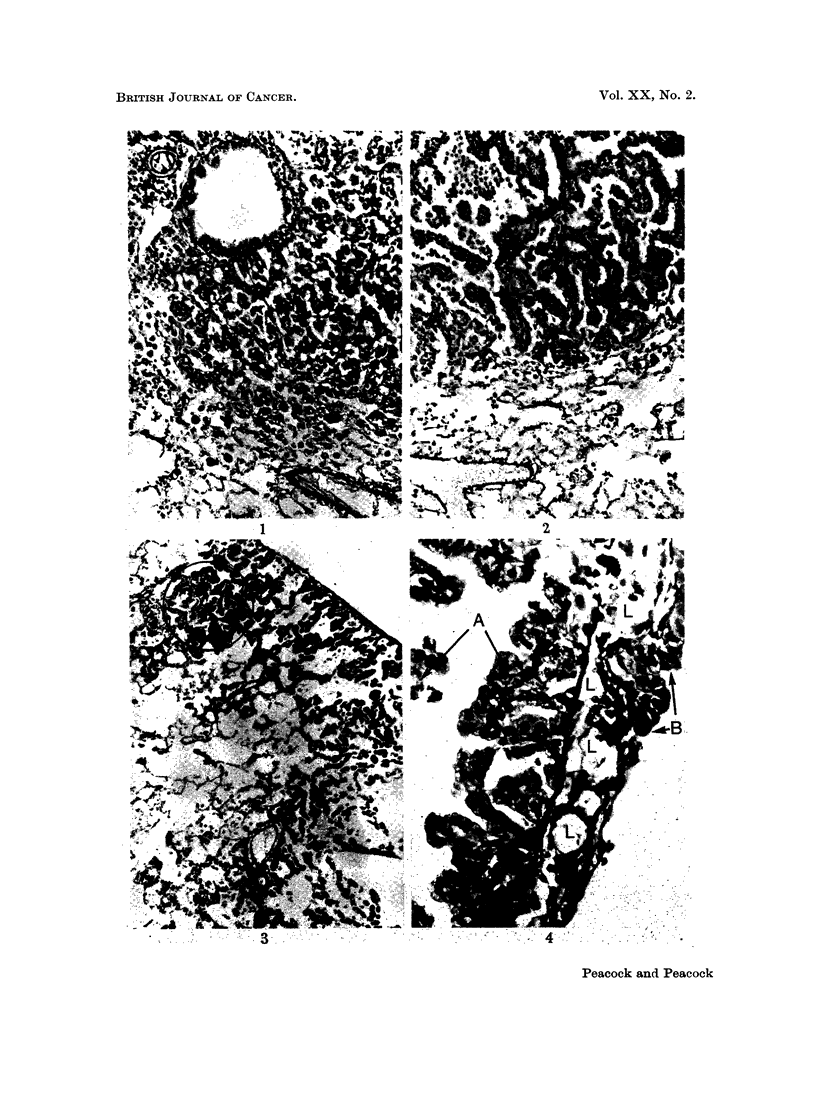

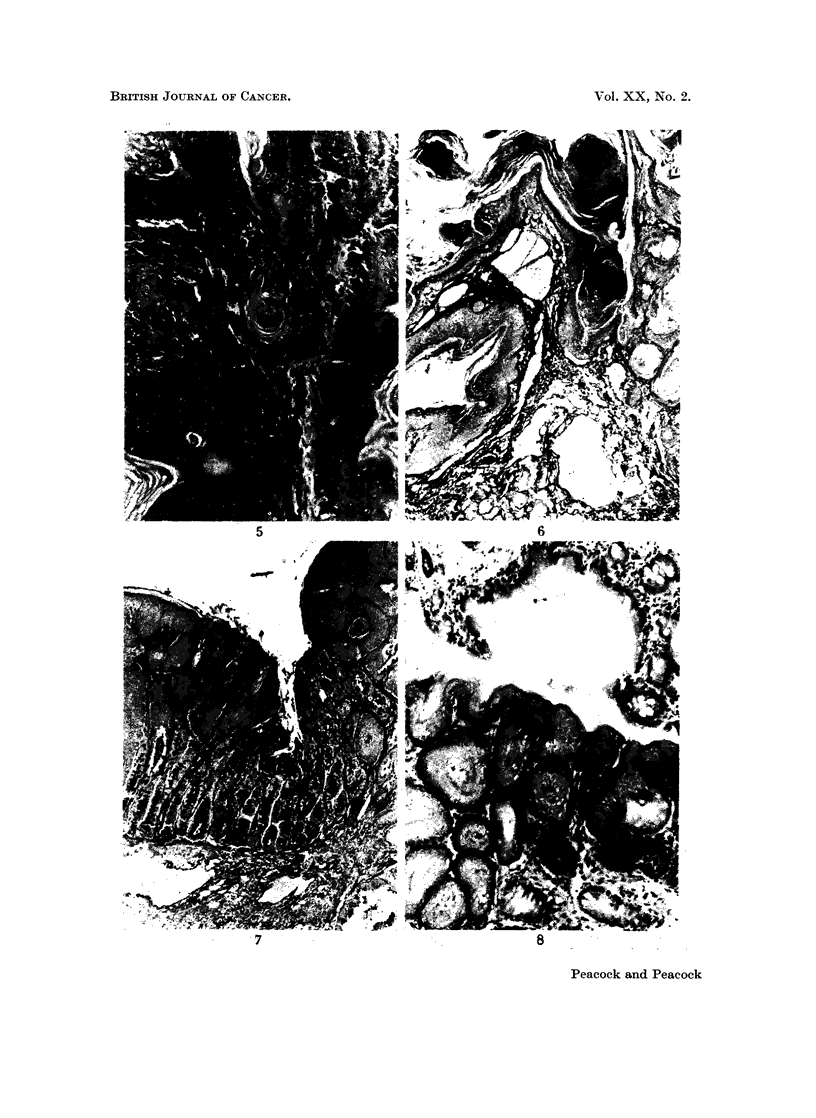

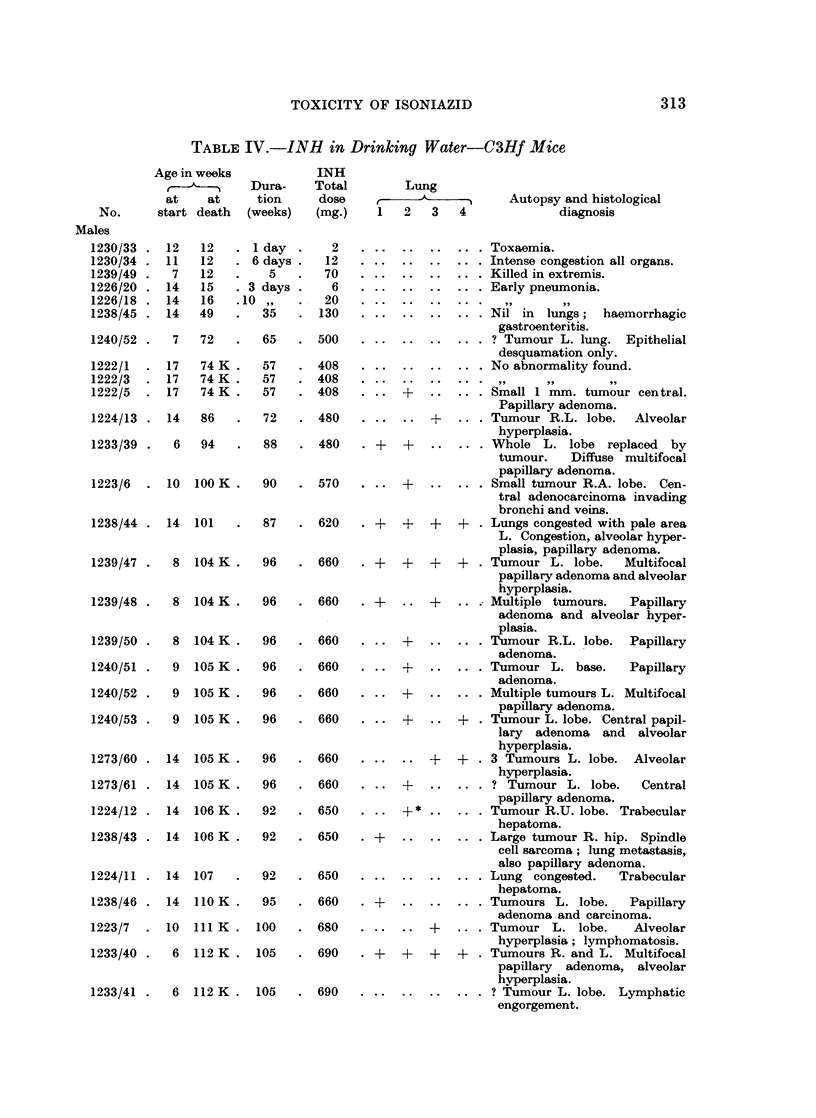

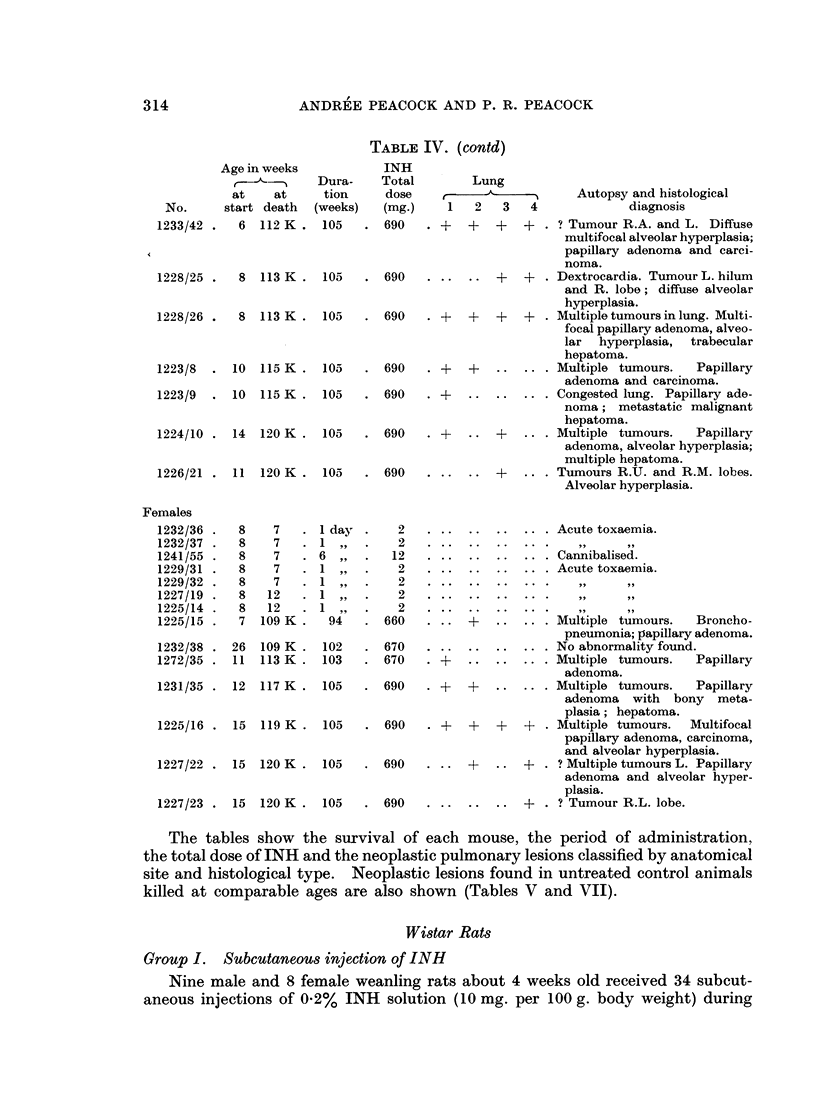

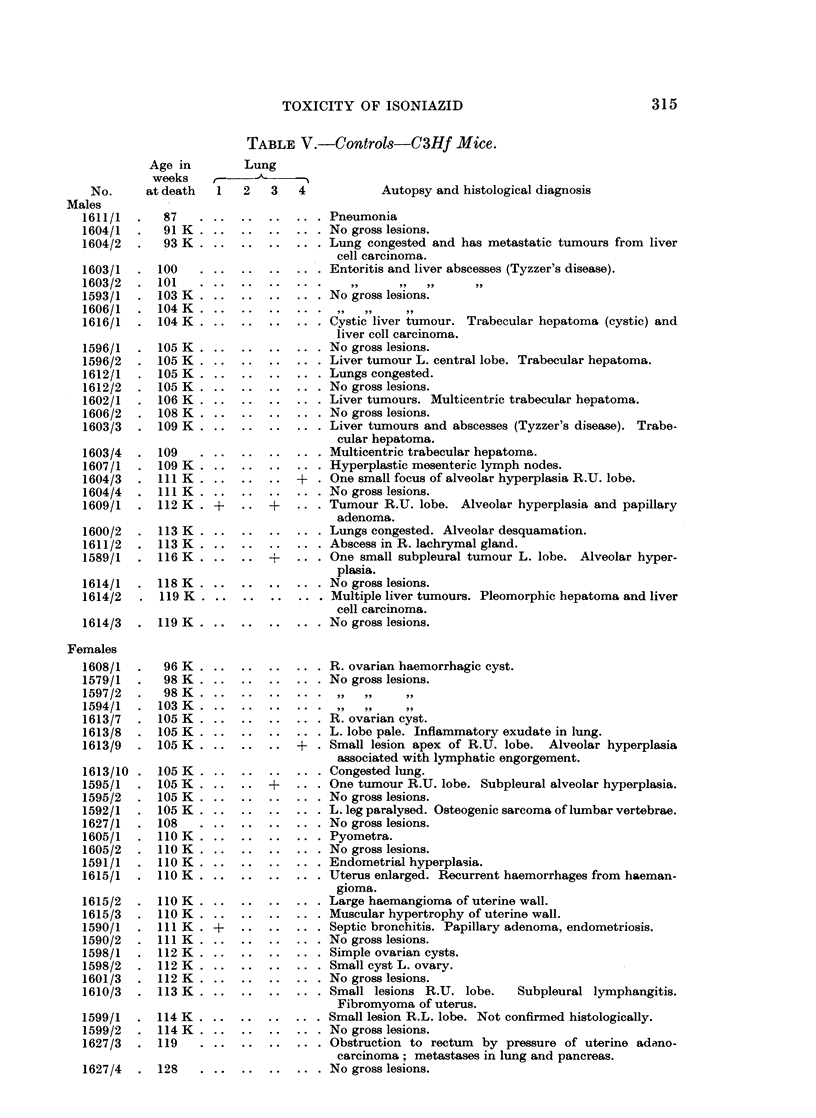

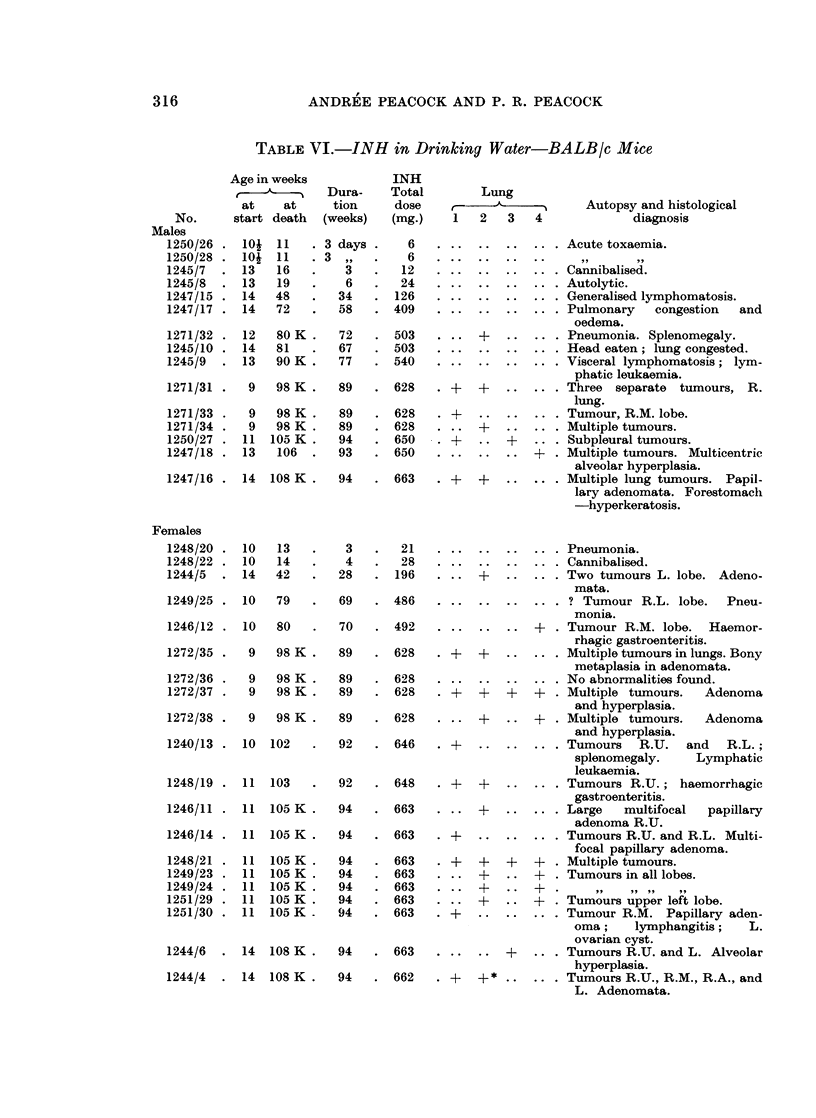

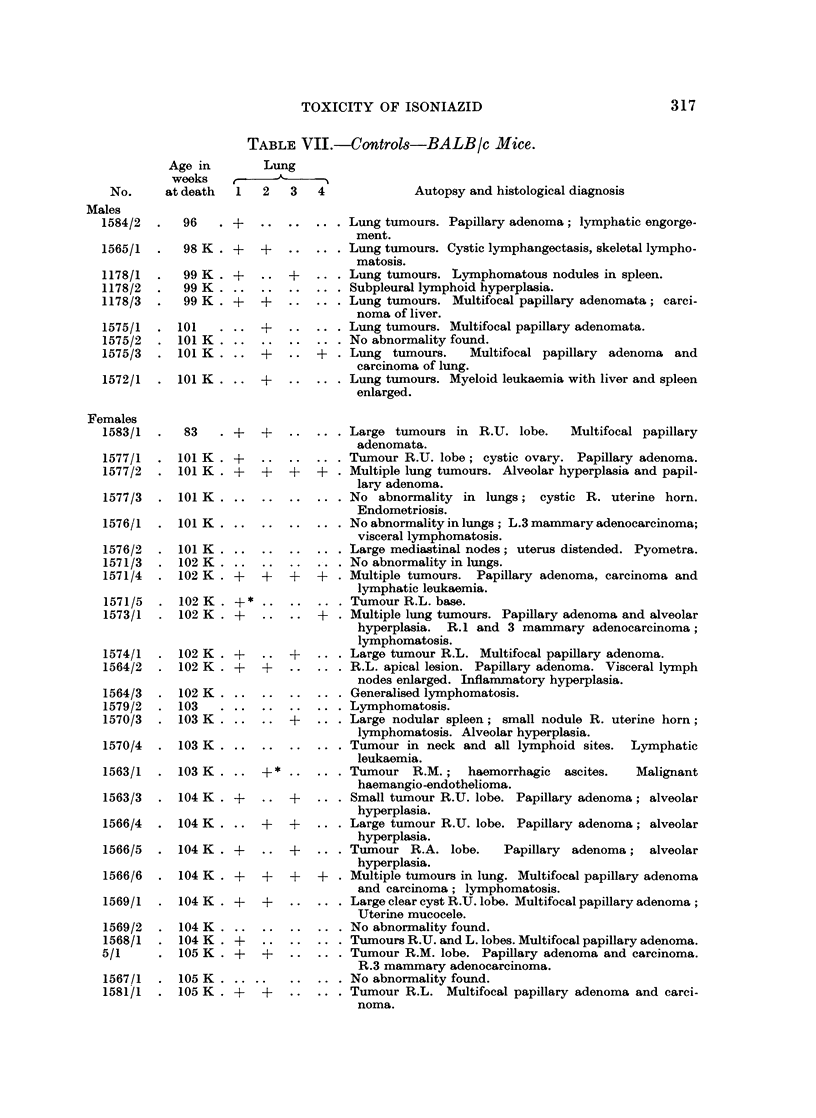

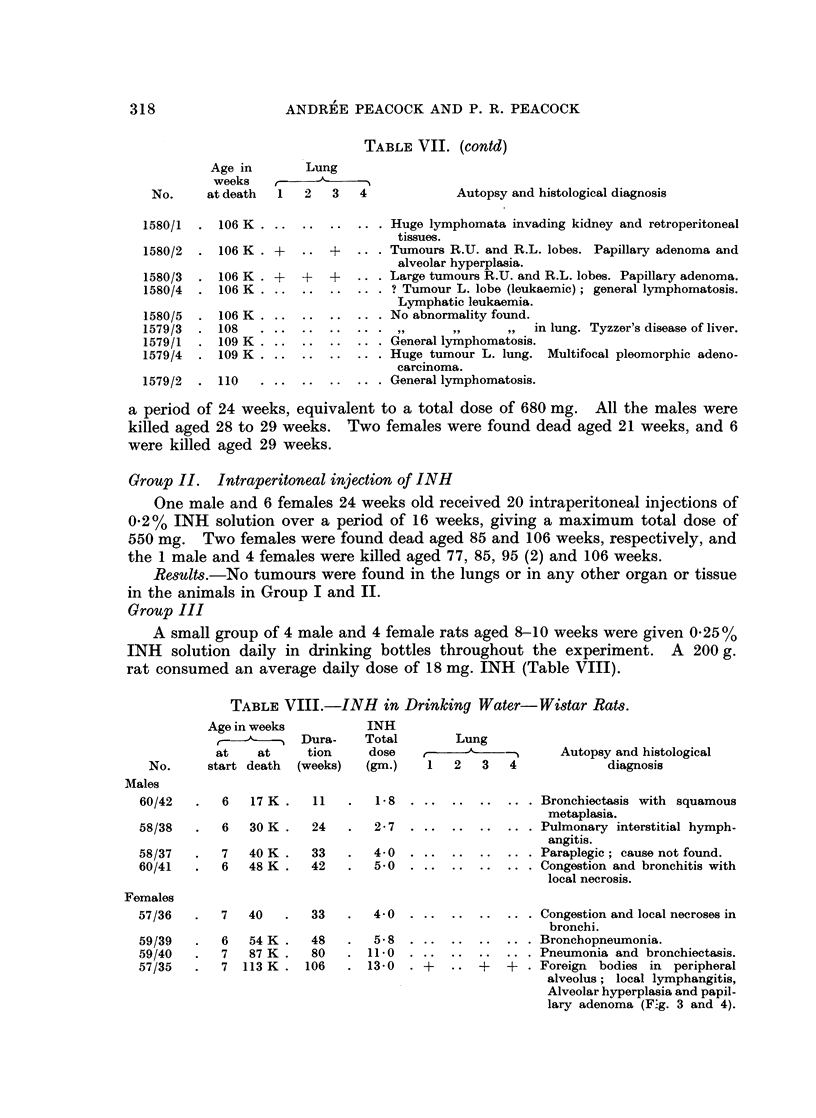

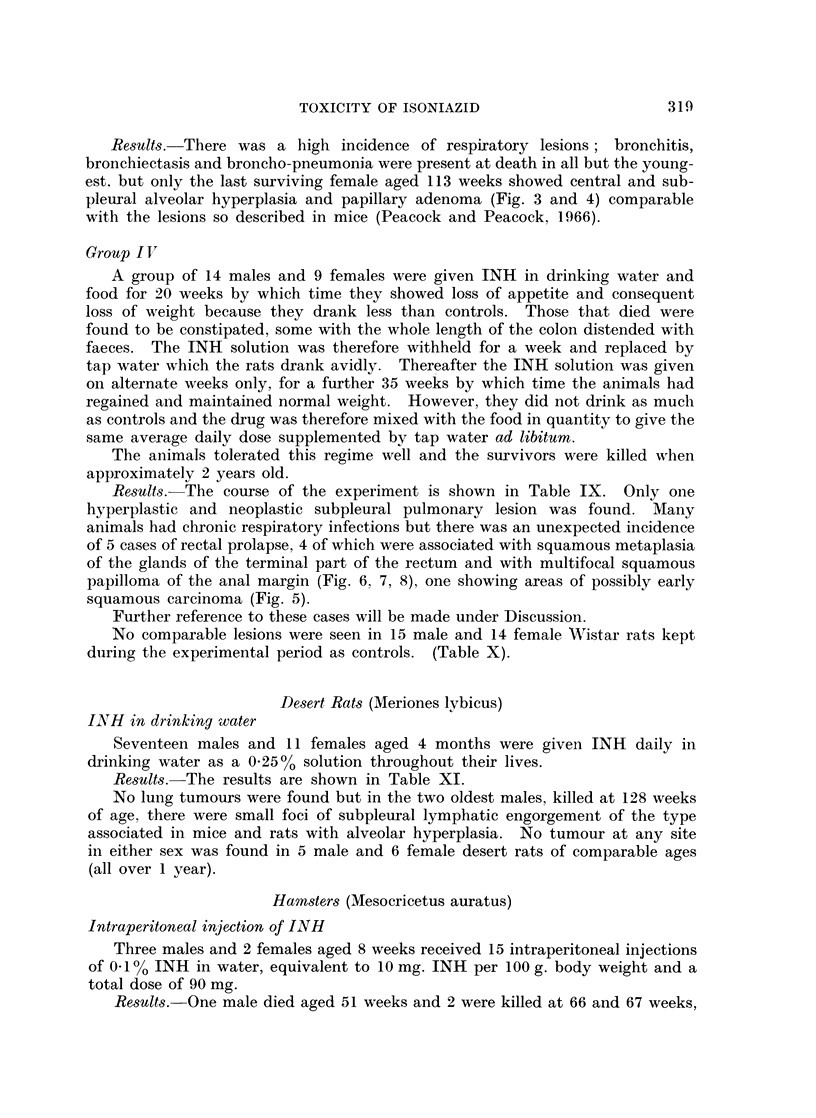

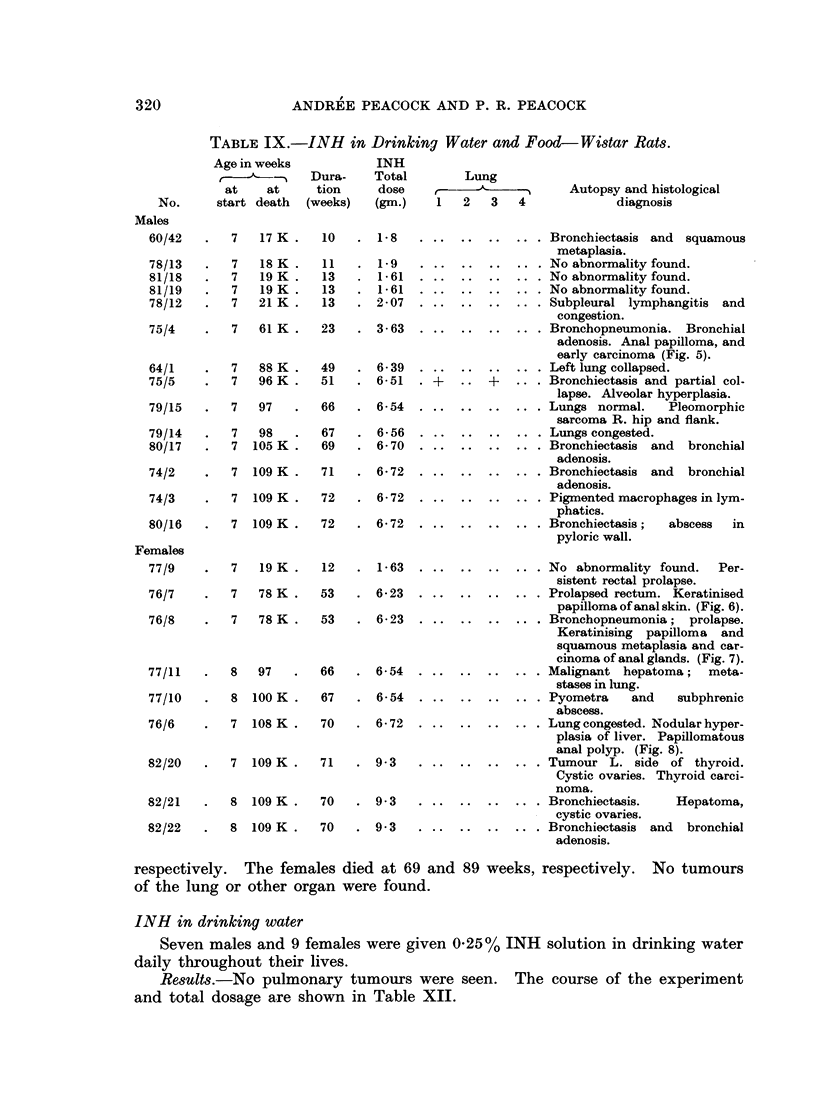

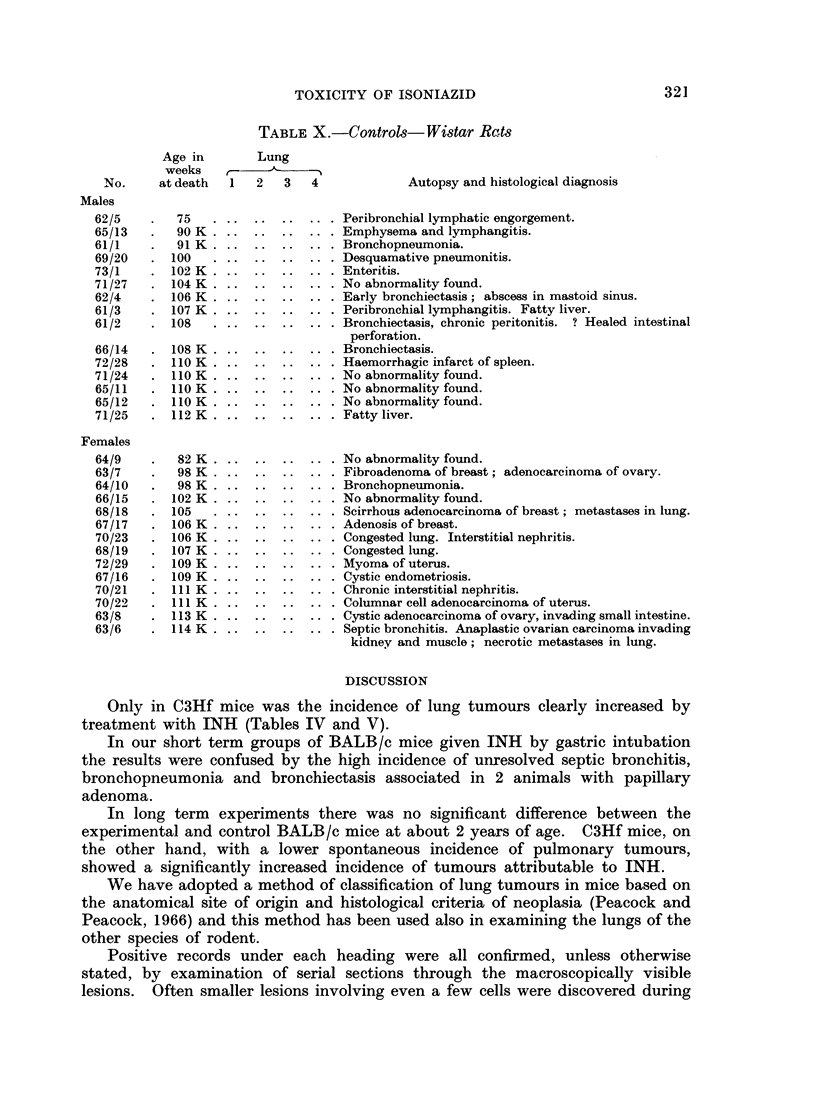

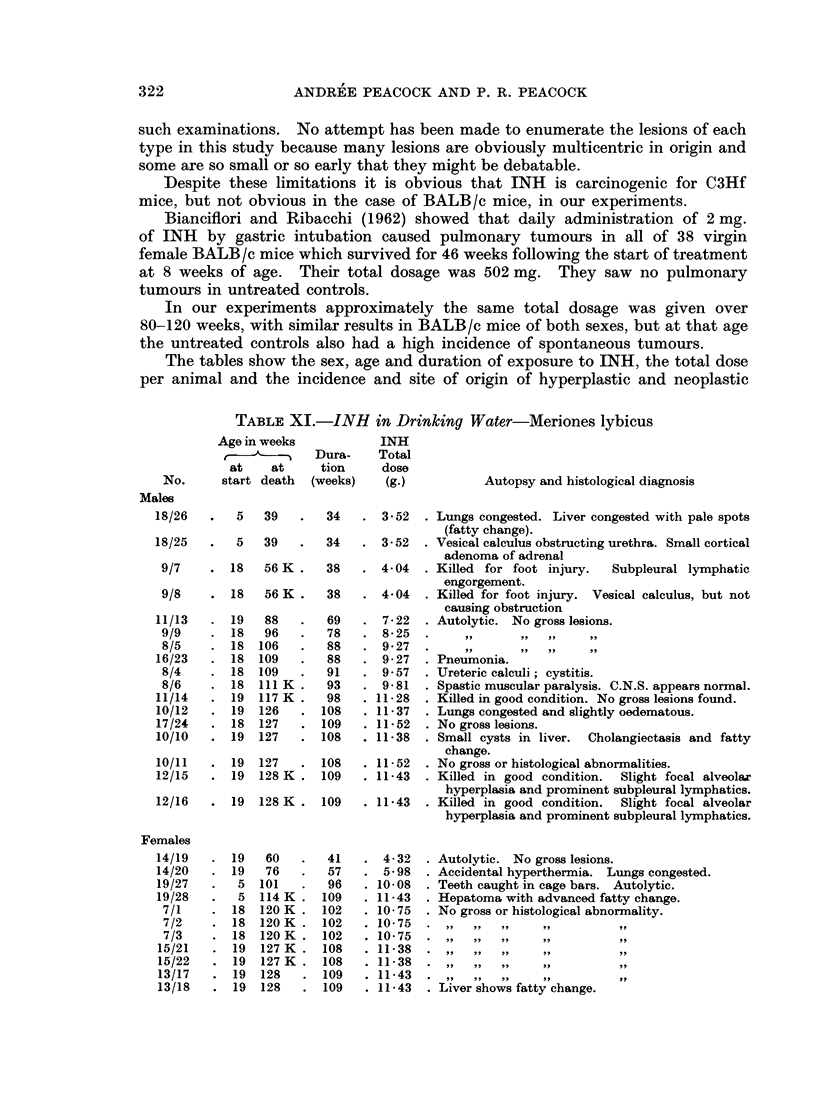

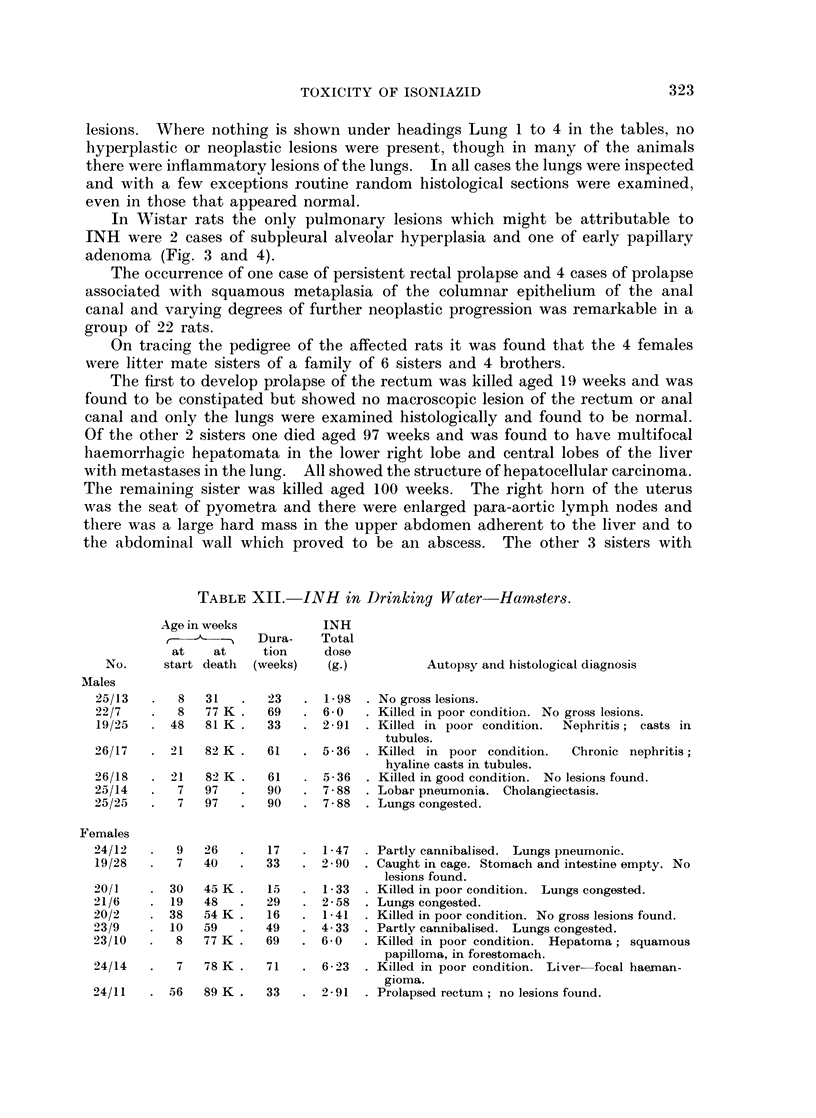

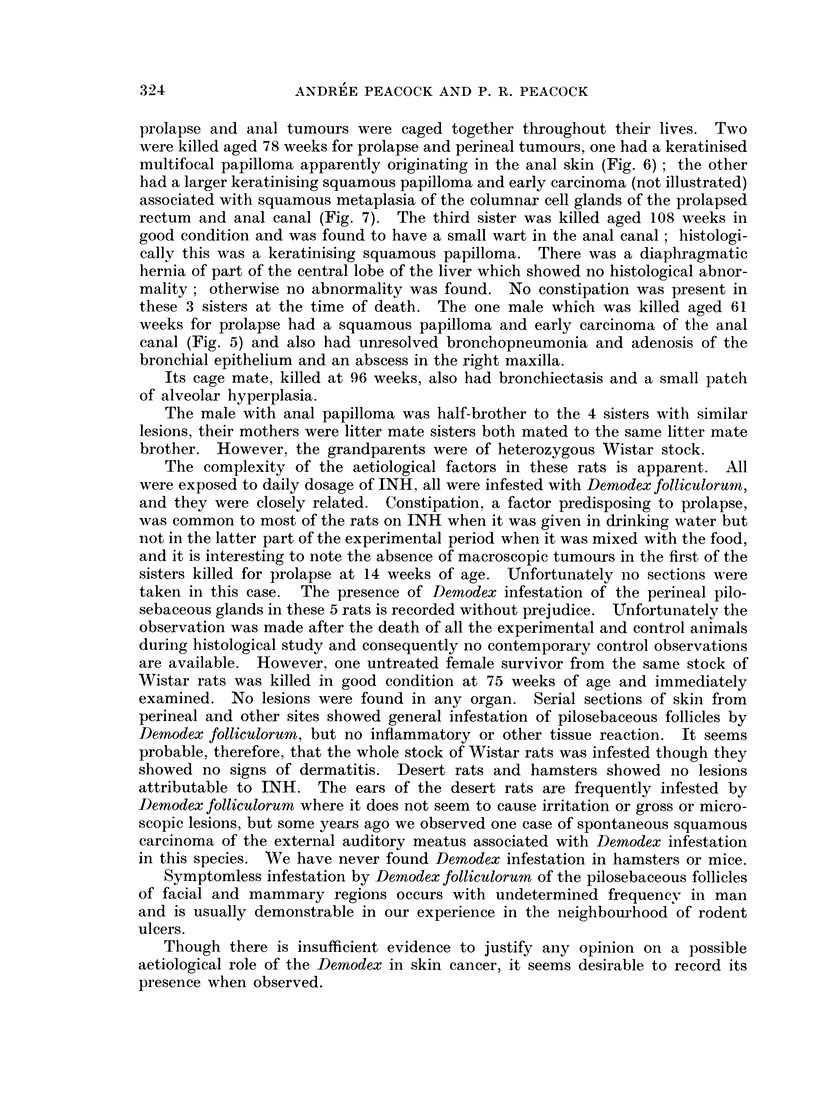

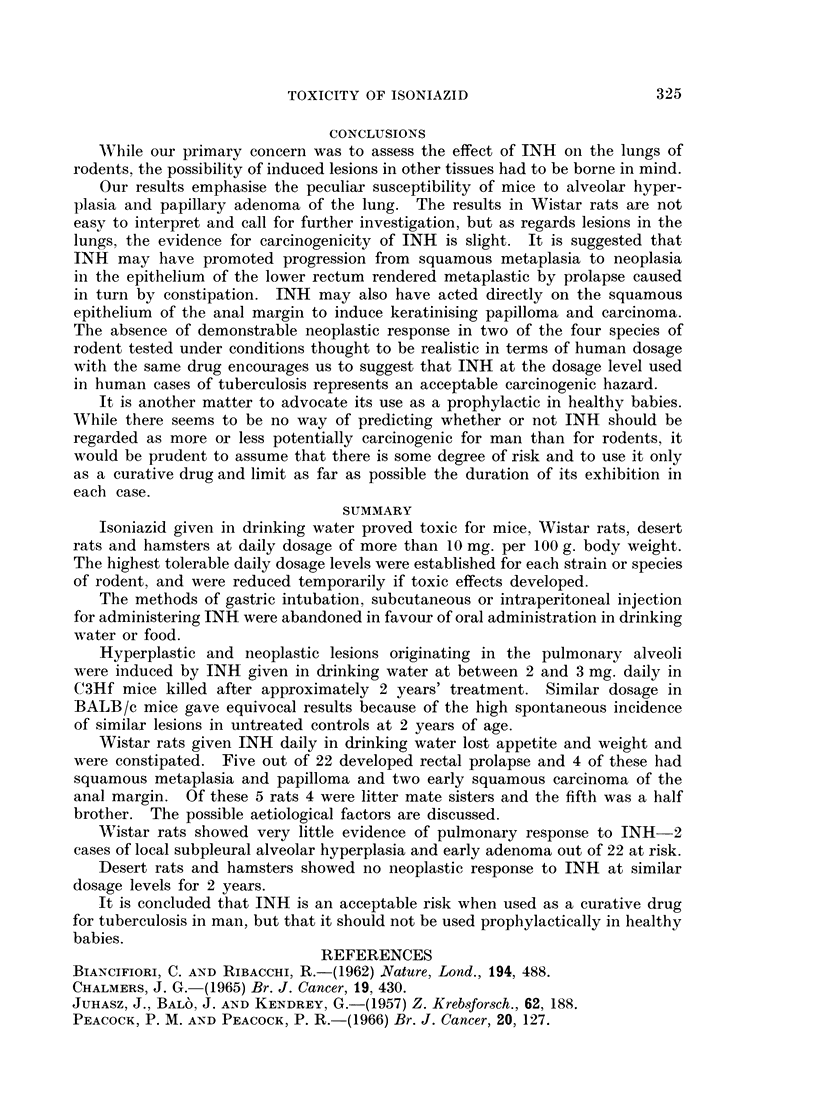


## References

[OCR_02498] BIANCIFIORI C., RIBACCHI R. (1962). Pulmonary tumours in mice induced by oral isoniazid and its metabolites.. Nature.

[OCR_02499] CHALMERS J. G. (1965). THE EFFECT OF ISONIAZID ON THE CLEARANCE OF PYRUVIC AND ALPHA-OXOGLUTARIC ACIDS IN THE URINE OF MICE, MERIONES LYBICUS AND RATS.. Br J Cancer.

[OCR_02499a] CHALMERS J. G. (1965). THE EFFECT OF ISONIAZID ON THE CLEARANCE OF PYRUVIC AND ALPHA-OXOGLUTARIC ACIDS IN THE URINE OF MICE, MERIONES LYBICUS AND RATS.. Br J Cancer.

[OCR_02501] JUHASZ J., BALO J., KENDREY G. (1957). Uber die geschwulsterzeugende Wirkung des Isonicotinsäurehydrazid (INH).. Z Krebsforsch.

